# Tungsten anchored onto functionalized SBA-15: an efficient catalyst for diastereoselective synthesis of 2-azapyrrolizidine alkaloid scaffolds†

**DOI:** 10.1039/c9ra02825k

**Published:** 2019-06-24

**Authors:** Javad Safaei-Ghomi, Atefeh Bakhtiari

**Affiliations:** Department of Organic Chemistry, Faculty of Chemistry, University of Kashan P. O. Box 87317-51167 Kashan I. R. Iran safaei@kashanu.ac.ir +98 31 55552935 +98 31 55912385

## Abstract

We used a novel hybrid catalyst in chemo-, regio-, and diastereoselective multi-component reactions (MCR) for the synthesis of the 2-aza analogue of pyrrolizidine and spirooxindole-2-azapyrrolizidine derivatives. The nanocatalyst, W(iv)/NNBIA–SBA-15 [where NNBIA = *N*,*N*′-(ethane-1,2-diyl)bis(2-aminobenzamide)] was synthesized by covalent grafting on chloro-functionalized SBA-15. The synthesis process was followed by the anchoring of WCl_6_ to catch the desired catalyst. The quality of the catalyst was assessed using different analytical techniques such as X-ray diffraction spectroscopy (XRD), Fourier-transform infrared spectroscopy (FT-IR), N_2_ adsorption analysis, transmission electron microscopy (TEM), field emission scanning electron microscopy (FESEM), energy-dispersive X-ray spectroscopy (EDX), ammonia Temperature Programmed Desorption (TPD), X-Ray photoelectron spectroscopy (XPS) and thermogravimetric, differential thermal analysis (TGA-DTA). The catalyst, W(iv)/NNBIA–SBA-15, with high catalytic performance is a good candidate for the diastereoselective synthesis of 2-azapyrrolizidine alkaloid scaffolds. The catalyst could be recovered for reuse without noticeable loss of activity.

## Abstract 2

We used a novel hybrid catalyst in chemo-, regio-, and diastereoselective multi-component reactions (MCR).

## Graphical abstract

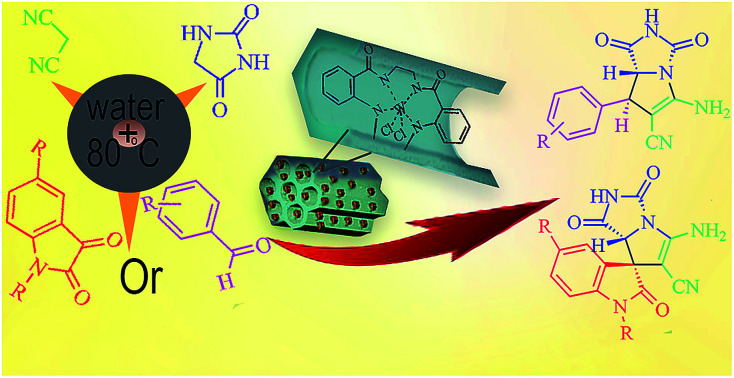

## 1. Introduction

Chemical researchers are focused on the synthesis of functionalized nitrogen and oxygen-containing organic compounds that have potential medicinal and biological activities.^1,2^ Diversity-oriented synthesis (DOS) is one of the strategies used to provide synthetic methods for the efficient combination of regiochemically and functionally diverse simple molecules.^3–5^ DOS is especially useful for those structures based on natural products or drug-like molecules.^6,7^ The aim of DOS is to achieve simultaneous, deliberate, and impressive syntheses of structurally diverse compounds such as drug molecules or natural products.^3–5,8^ MCRs^9^ can be placed in the class of DOS chemistry due to green characteristics, straightforward reaction designs, high degrees of atom economy, lower costs and environmentally friendliness.^10^ The use of water in chemical reactions has many advantages in the field of green chemistry, such as modifying the reactivity and selectivity of the reaction.^11^

Spirocyclic structures that contain one sp^3^ carbon atom common to two rings have many biological properties. They have attracted the considerable attention of organic chemists because of being in natural ingredients.^12^

One of the most important classes of biological compounds is hydantoins.^13–15^ The presence of three disparate nucleophilic centers in hydantoin provides the ability to synthesize many biologically active molecules from several alternative cyclization pathways.^16^ Pyrrolizidine alkaloids consisting of two fused five-member carbon rings with a nitrogen atom at one bridgehead belong to the aza-heterocyclic family.^17^ They are commonly used in chemical biology and medicinal chemistry due to their medicinal properties.^18,19^ The presence of a nitrogen-substituted carbon in the stereogenic center of pyrrolizidine alkaloids increases their biological properties. Some of their derivatives such as retronecine,^20^ heliotridine,^21^ and platynecine^22^ show anti-tumoural, anti-microbial, and anti-viral effects. In previous reports, conventional organic catalysts such as NEt_3_ (ref. 23) and piperidine^16,24^ were employed for synthesizing 2-azapyrrolizidine alkaloid scaffolds.

Tungsten is a transition metal of Group VIb of the periodic table of elements, and it has attracted a great deal of attention due to its extraordinary properties.^25^ The use of tungsten in new applications has made it useful as an essential commodity in recent years.^26^ Tungsten based catalysts with unique functional properties have been widely used for various applications, such as the selective oxidation of unsaturated compounds,^27^ metathesis and isomerization of alkenes^28^ and the dehydrogenation of alcohols.^29^ They are most useful for addition reactions to olefinic double bonds.^30^ Recently, researchers have attempted to insert tungsten into siliceous mesoporous molecular sieves (*e.g.*, silica, M41S, and SBA-*n*)^31–33^ by various methods such as grafting,^34^ cogelation,^35^ impregnation^36^ or insertion.^37^ The diverse compositions of incorporated tungsten into siliceous mesoporous materials have resulted in improved catalytic activities.

Considerable attention has been paid to designing heterogeneous catalysts, due to their advantages such as easy separation, easy recovery, and high reusability. The surface support has an influential effect on the behavior of heterogeneous catalysts. Several solid supports have been introduced such as polymer resins, silica, alumina, silica-coated magnetic particles, and mesoporous molecular sieves. The fully ordered structure of mesoporous silica leads to the generation of a framework with regular porosity, so ligands could be immobilized well on it. Because of the advantages of mesoporous silica, it could be vastly employed in separation, catalysis, gas storage, drug delivery, and biomolecule applications. Since the development of ordered mesoporous silicas,^38,39^ SBA-15 materials developed by Zhao and Stucky^40,41^ serve in many fields due to their interesting textural properties such as their appreciable thermal and hydrothermal stabilities, large pore volumes, uniform-sized pores (in the range 4–30 nm) and high specific surface areas (above 1000 m^2^ g^−1^).^42–48^ The creation of organic–inorganic hybrid catalysts is the best methodology to overcome a lack of functionality by using a variety of organic functional groups.^49–51^ The unique abilities of organic–inorganic hybrid materials make these systems highly attractive candidates for a range of applications including recyclable catalysts, drug delivery, biotechnology, biomedicine *etc*.^52–54^ There are a number of methods for the synthesis of hybrids catalyst. Among them, stable attachment and less leaching^55^ are observed post-synthetically. The available ligand precursors can be used for multifunctional integration of SBA-15.

**Scheme 1:**
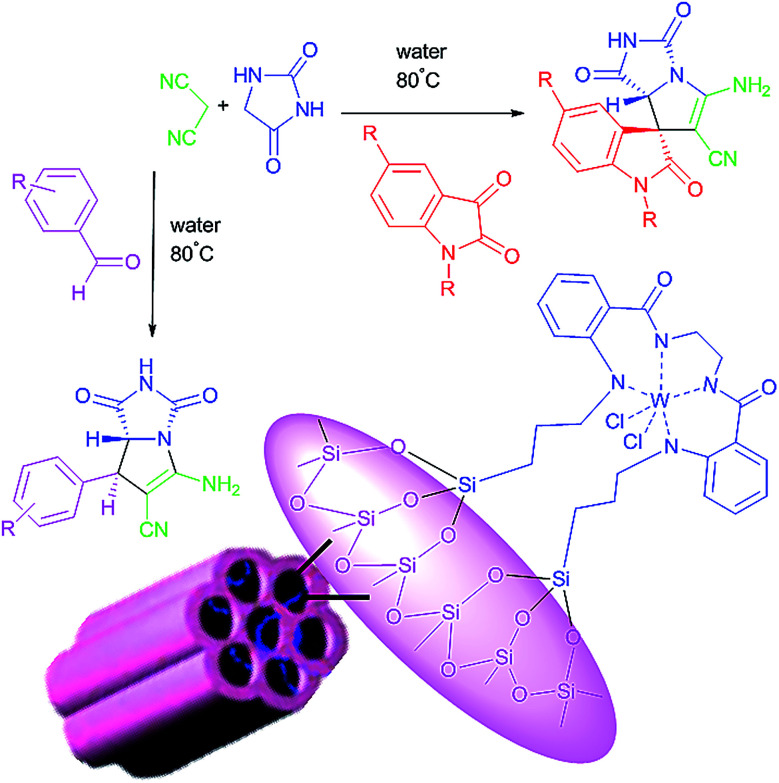
Synthesis of the 2-aza analogue of pyrrolizidine and spirooxindole-2-azapyrrolizidine derivatives.

We are interested in the development of our research to prepare more effective catalysts to synthesize a 2-aza analogue of pyrrolizidine and spirooxindole-2-azapyrrolizidine derivatives. The nanocatalyst, W(iv)/NNBIA–SBA-15, was prepared by applying simple and cost-effective materials and also it was characterized as a new inorganic–organic hybrid catalyst. The one-pot reactions of aldehyde or isatin (1 mmol), malononitrile (1 mmol), and hydantoin (1 mmol) were catalyzed by W(iv)/NNBIA–SBA-15 in water (Scheme 1).

## 2. Results and discussion

### 2.1. Structural analysis of W(iv)/NNBIA–SBA-15

**Fig. 1:**
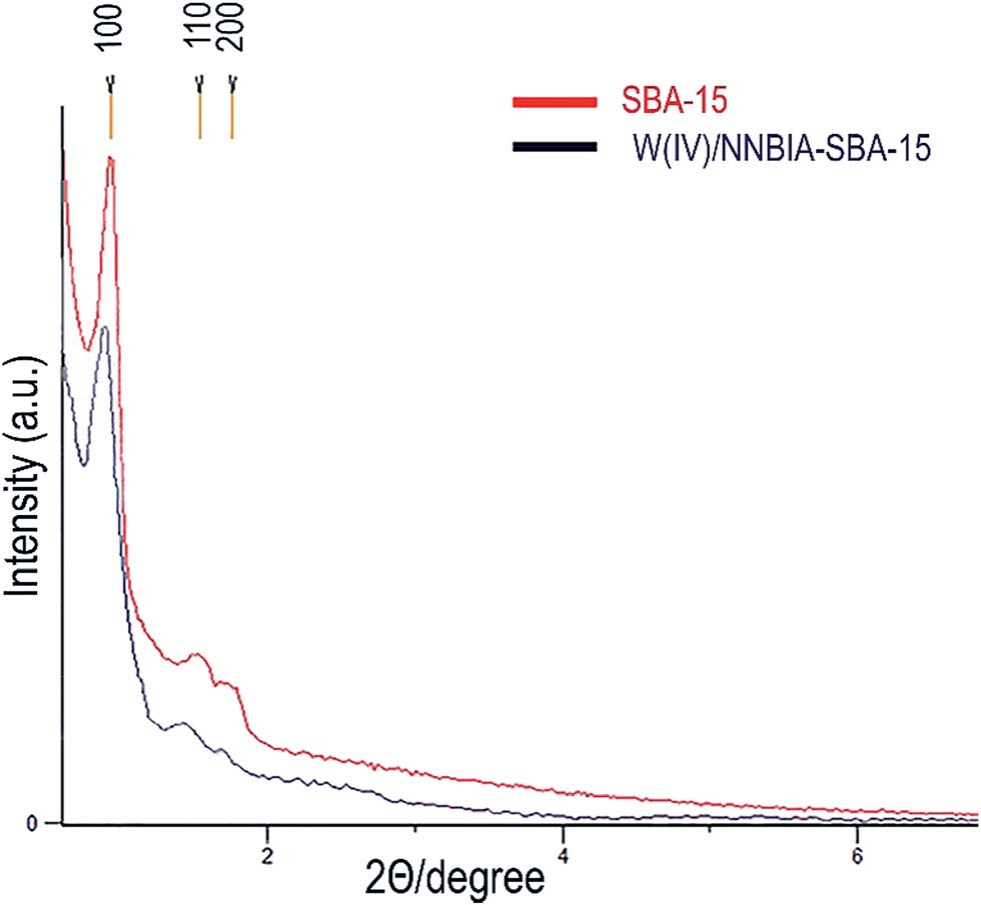
The XRD patterns of SBA-15 and W(iv)/NNBIA–SBA-15.

A small angle X-ray diffraction method was used to monitor the effect of incorporation of the ligand, NNBIA, and the metal on the framework of SBA-15 (Fig. 1). The patterns for both of SBA-15 and W(iv)/NNBIA–SBA-15 show an intense diffraction peak (100) which references to the mesostructure with a remarkable degree of long-range ordering. The observed intensities of the two secondary peaks corresponding to (110) and (200) reflections are weak and they are attributed to the 2D-hexagonal planes of the mesoporous structure.^56,57^ The XRD pattern of W(iv)/NNBIA–SBA-15 indicates a striking effect by loading of the ligand and the metal. The width and intensity of the diffraction peak (100) become broader and weaker respectively, and also it shifts to lower 2*θ* values than the XRD pattern of pure SBA-15. Partial blockage of the available pores of the anchored catalyst decreased the long-range order of SBA-15.^58^ It can be inferred from the above results that the framework of SBA-15 remains perfect after incorporation of W(iv)-NNBIA.

**Fig. 2:**
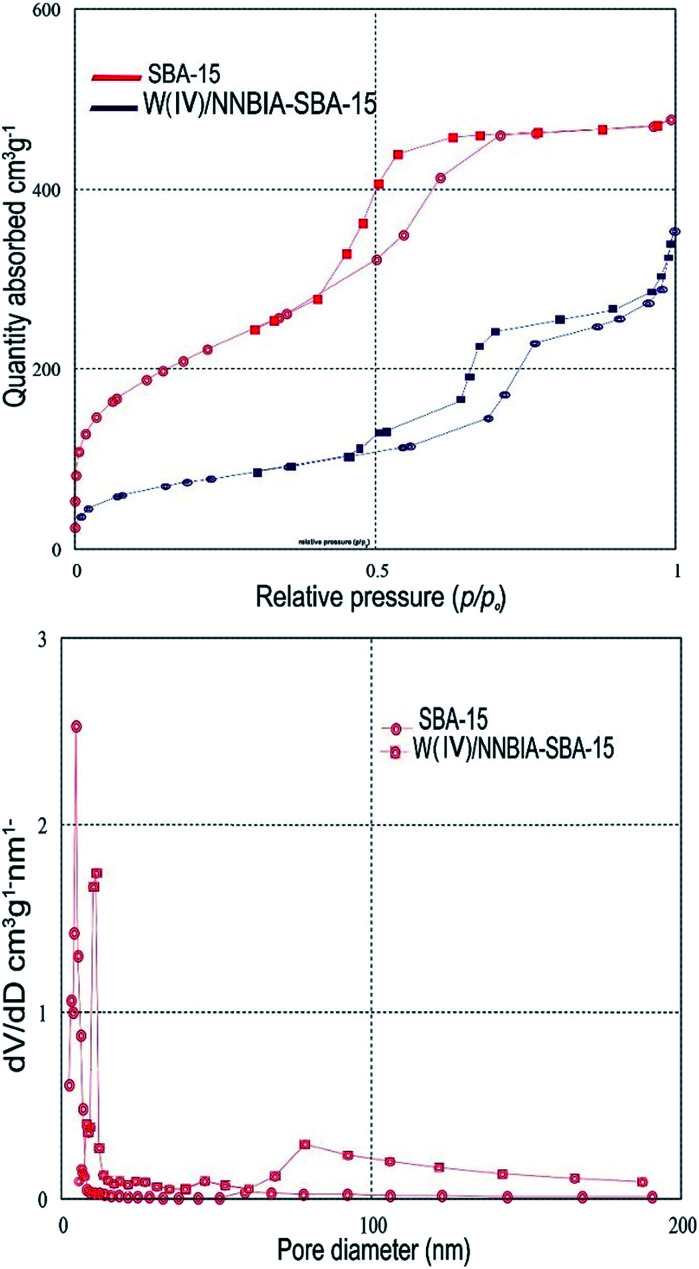
N_2_ adsorption–desorption isotherms and BJH pore size distributions of SBA-15, and W(iv)/NNBIA–SBA-15.

The surface and structure properties of pure SBA-15 and W(iv)/NNBIA–SBA-15 were evaluated by N_2_ adsorption–desorption isotherms analysis and BJH pore size distributions (Fig. 2). Both of them show a type IV isotherm with an H1 hysteresis loop which is in agreement with the typical mesostructure. The steep adsorption step at 0.4 to 0.8 *P*/*P*_o_ corresponds to the capillary condensation of nitrogen in uniform pores. The sharpness of the pore-filling step in the adsorption and desorption curves is related to the uniformity of the mesopore size distribution. Due to pore blocking effect, the functionalization of SBA-15 not only shifts the inflection to a lower *P*/*P*_o_ range but also diminishes the sharpness of it.^43^ The reduction in sharpness of the functionalized SBA-15 is due to less uniformity in the mesopore size distribution. The above results clearly indicate that the ordered mesoporous structure of SBA-15 with a narrow pore size distributions remains well after immobilizing W(iv)/NNBIA. The BET surface area (758 m^2^ g^−1^), the average pore diameter (9.4 nm) and the total pore volume (0.737 cm^3^ g^−1^) of SBA-15 were recorded (Table 1). The textural parameters of W(iv)/NNBIA–SBA-15 are changed by the cooperation of the ligand and the metal. The data for the surface area, average pore width, and total pore volume for W(iv)/NNBIA–SBA-15 are presented in Table 1. The results show that W(iv) is placed mainly inside the channels of the modified SBA-15 materials.

**Table 1:** Structural and textural parameters of SBA-15, and W(iv)/NNBIA–SBA-15

Sample	*S*_BET_ a [cm^2^ g^−1^]	*D*_p_ b [nm]	*V*_P_ c [cm^3^ g^−1^]
SBA-15	758.68	9.40	0.737
W(iv)/NNBIA–SBA-15	182.29	8.06	0.392

a *S*_BET_ = surface area.

b *D*_p_ = average pore width.

c *V*_p_ = total pore volume.

**Fig. 3:**
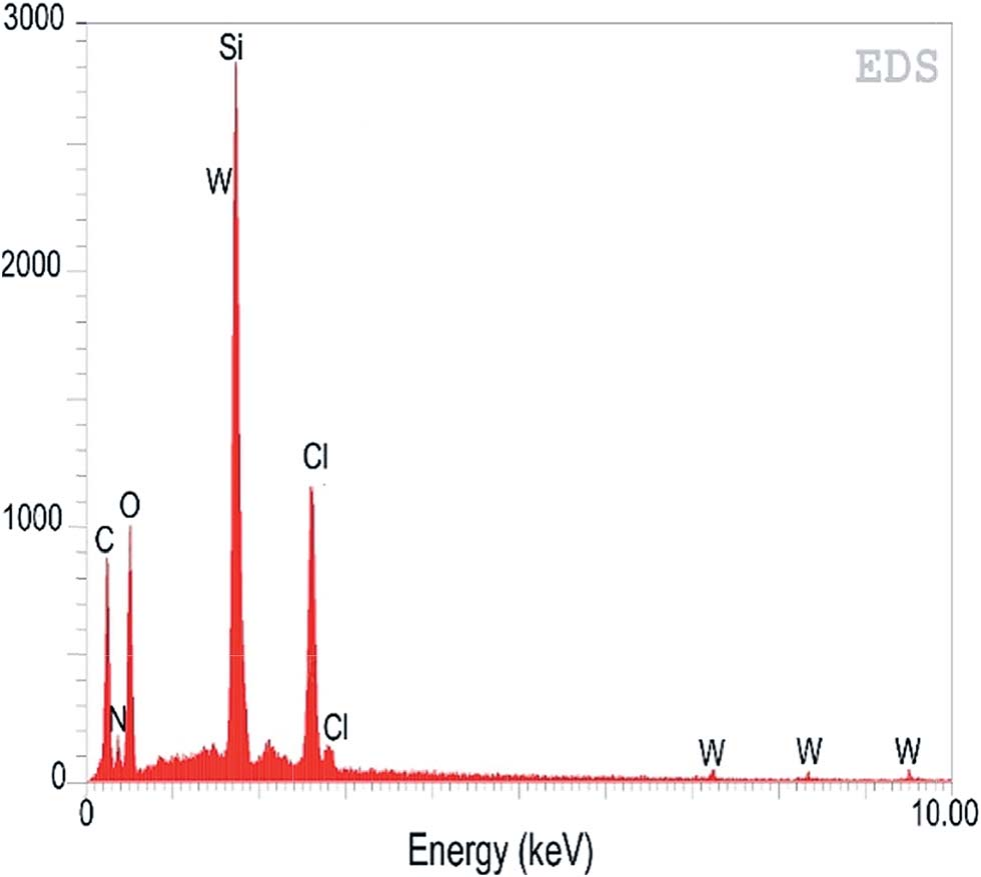
EDS spectrum of W(iv)/NNBIA–SBA-15.

The incorporation efficiency and contents of the ligand and metal in the mesoporous SBA-15 were analysed using an EDS spectrum (Fig. 3). The amount of carbon, nitrogen, oxygen, silicon, chlorine and tungsten in W(iv)/NNBIA–SBA-15 were calculated to be 30.63, 15.06, 30.44, 9.89, 8.39, 5.59 (wt%), respectively. The EDS mapping analysis suggested a highly uniform dispersion of the ligand, NNBIA and tungsten on SBA-15.

**Fig. 4:**
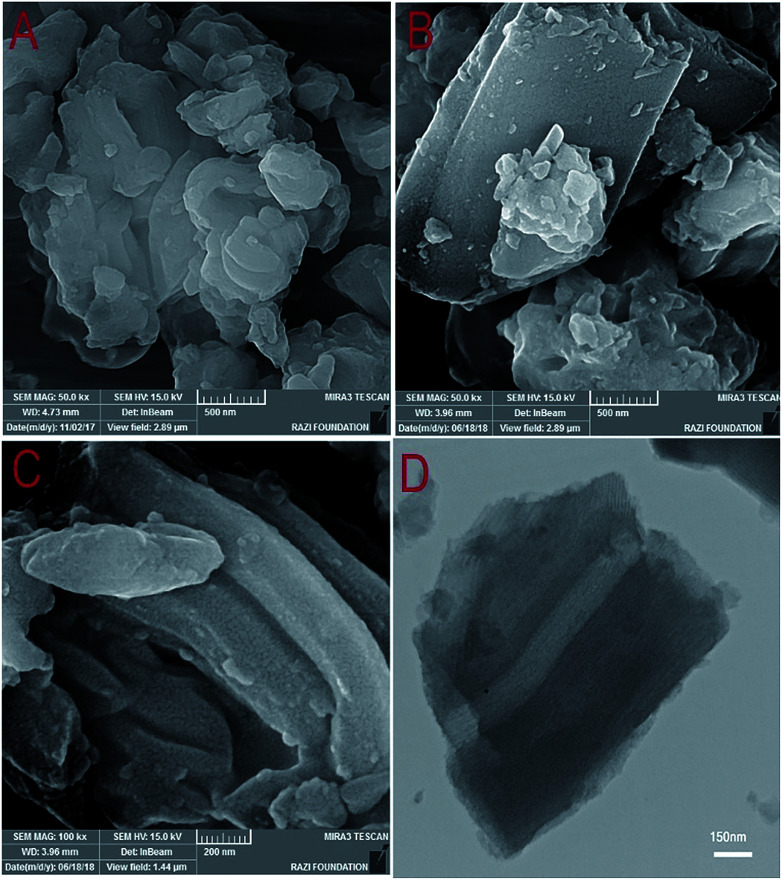
FESEM images of (A) SBA-15, (B) W(iv)/NNBIA–SBA-15 and (C) the used W(iv)/NNBIA–SBA-15 and (D) A TEM image of W(iv)/NNBIA–SBA-15.

The FESEM images of the SBA-15 and W(iv)/NNBIA–SBA-15 are shown in Fig. 4a and b. The FESEM images show that both of them have good structural integrity and morphology. The surface morphology was not changed by functionalization. The evaluation of the used catalyst structure by FESEM evidences that the morphology of the catalyst remained unchanged after the 5th cycle (Fig. 4c). This is the feasible reason for the extreme stability of the catalyst. The presence of a 2D hexagonal network was confirmed by the TEM image of a W(iv)/NNBIA–SBA-15 sample (Fig. 4d). The TEM image shows highly dispersed W(iv)/NNBIA both inside and outside the channels of SBA-15.

**Fig. 5:**
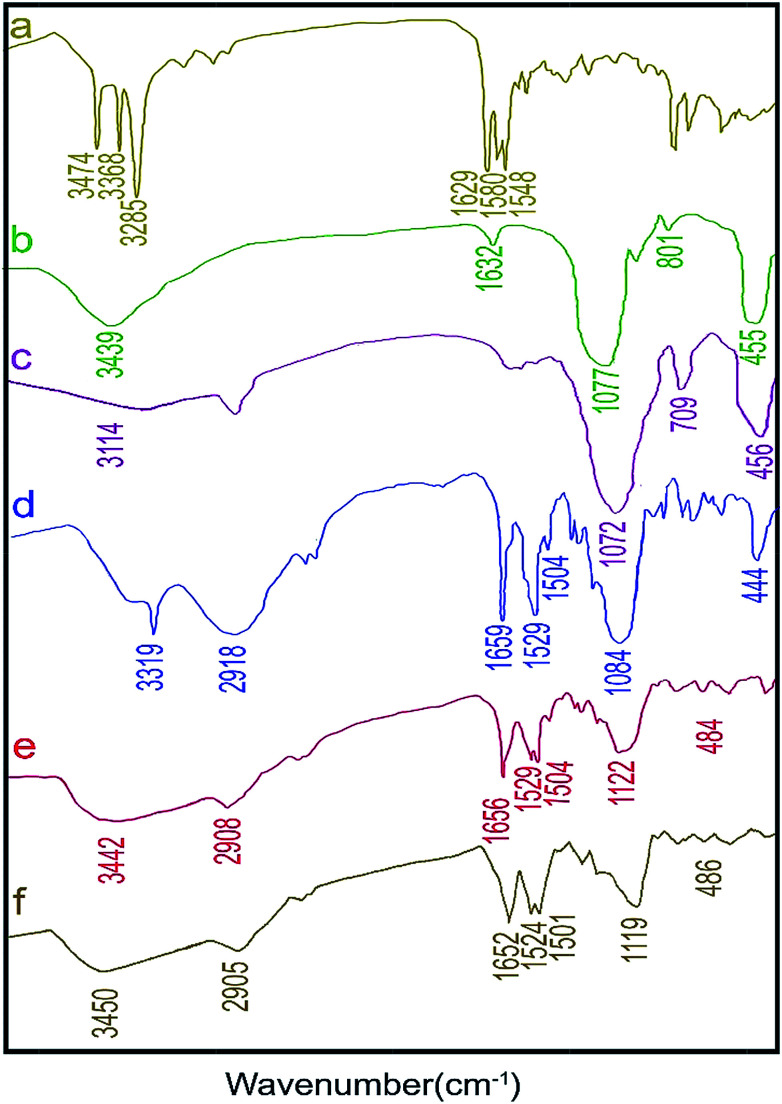
FT-IR spectra of (a) the ligand, NNBIA, (b) calcined SBA-15, (c) chlorofunctionalized SBA-15, (d) NNBIA–SBA-15, (e) fresh W(iv)/NNBIA–SBA-15 and (f) used W(iv)/NNBIA–SBA-15.

FT-IR spectroscopy is an effective technique to confirm and identify the structure of a catalyst. Fig. 5 shows the FT-IR spectra at all stages of the process of creating the catalyst. The peaks of the absorption bands associated with the formation of a condensed silica network appear around 1077, 801, and 455 cm^−1^. The peak at 1077 cm^−1^ can be attributed to the asymmetric stretching vibration peak of Si–O–Si groups. The peaks at 3439 and 1632 cm^−1^ are due to the stretching and bending vibrations of the surface hydroxyl groups, respectively.^48^ The spectrum of chloro-functionalized SBA-15 shows a peak at 3114 cm^−1^ related to a C–H stretching vibration within the propyl group, and the peak at 709 cm^−1^ corresponds to the C–Cl bonds. The FT-IR spectrum of the ligand, NNBIA, shows three strong absorption peaks due to the carbonyl groups and aromatic rings at 1548, 1580 and 1629 cm^−1^. Three other peaks at 3285, 3368 and 3474 cm^−1^ could be assigned to the N–H bonds of the ligand. The absorption peaks of the carbonyl groups are shifted toward low frequencies with the immobilization of the ligand, NNBIA, over the Cl–SBA-15. Due to ligand binding, the peak of the N–H bond was observed at 3319 cm^−1^ and the C–Cl stretch disappeared. Coordination of WCl_6_ to NNBIA–SBA-15 led to the disappearance of a sharp absorption peak at 3319 cm^−1^ that could be attributed to the removal of the N–H bonds. A wide peak at 3442 cm^−1^ could be attributed to some unreacted hydroxyl groups remaining on the surface of SBA-15. The combination of the organic ligand with the W is confirmed by these results. The reused catalyst after five runs had no obvious changes in structure, based on the results of the FT-IR spectrum when compared with the spectrum of the fresh catalyst.

**Fig. 6:**
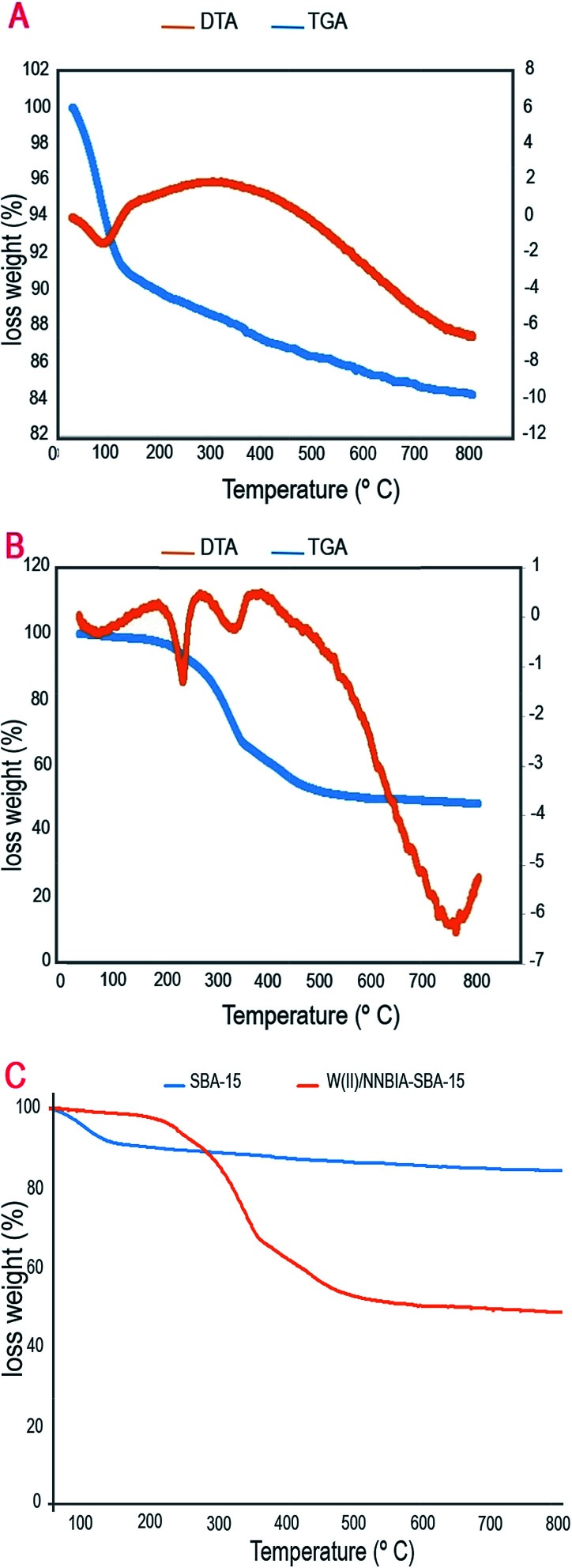
TGA and DTA curves of (A) SBA-15, and (B) W(iv)/NNBIA–SBA-15 and (C) TGA curves of SBA-15 and W(iv)/NNBIA–SBA-15.

The thermal behaviors of SBA-15 and W(iv)/NNBIA–SBA-15 from 30 °C to 800 °C are represented in Fig. 6. The TGA profile of the silica support exhibits two steps of weight loss. The first weight loss (∼9%) is in the region from ∼150 °C to 250 °C and corresponds to a sharp visible exothermic peak in the DTA analysis in the same temperature region. This is related to the loss of physically bound water and the break-up of a hydrogen bonded network. The second region is from ∼200 °C to 800 °C and is associated to the dihydroxylation of OH groups. It can be evidently supported by one strong exothermic peak in the same temperature range in the DTA analysis. The TGA profile of W(iv)/NNBIA–SBA-15 shows a weight loss of 36% in the temperature range of 350–600 °C, which is accompanied by a broad exothermic peak between 370 °C and 750 °C in the DTA curve. This is associated with ligand desorption or decomposition. Based on these results, the amount of active catalyst present on the heterogeneous SBA support is 1.5 mmol. The decomposition of the W(iv)/NNBIA complex is done at a high temperature, and that demonstrates the high thermal stability of the complex. Consequently, the results reported thus far indicate that W(iv)/NNBIA is formed and principally located, inside the SBA-15 pore channels.

**Fig. 7:**
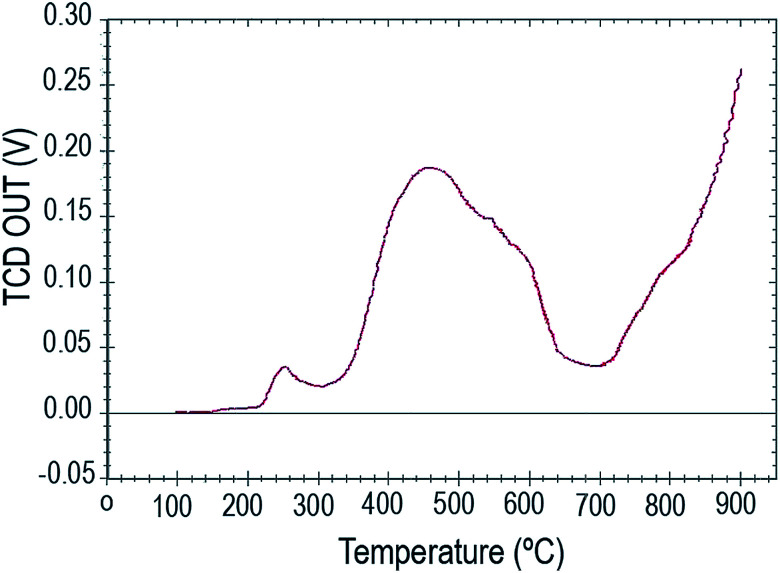
NH_3_-TPD spectrum of W(iv)/NNBIA–SBA-15.

The NH_3_-TPD profile of W(iv)/NNBIA–SBA-15 is shown in Fig. 7. The acid sites are distributed in two temperature regions. The second peak at about 450 °C is sharper and wider than the first one at about 250 °C. They could be attributed to weak acid sites and medium-strong acid sites, respectively. The desorption peak at the low temperature of 250 °C was observed due to the physically adsorbed ammonia and weakly bonded ammonia. The second-wide peak could be assigned to ammonia on the medium-strong acid sites.

**Fig. 8:**
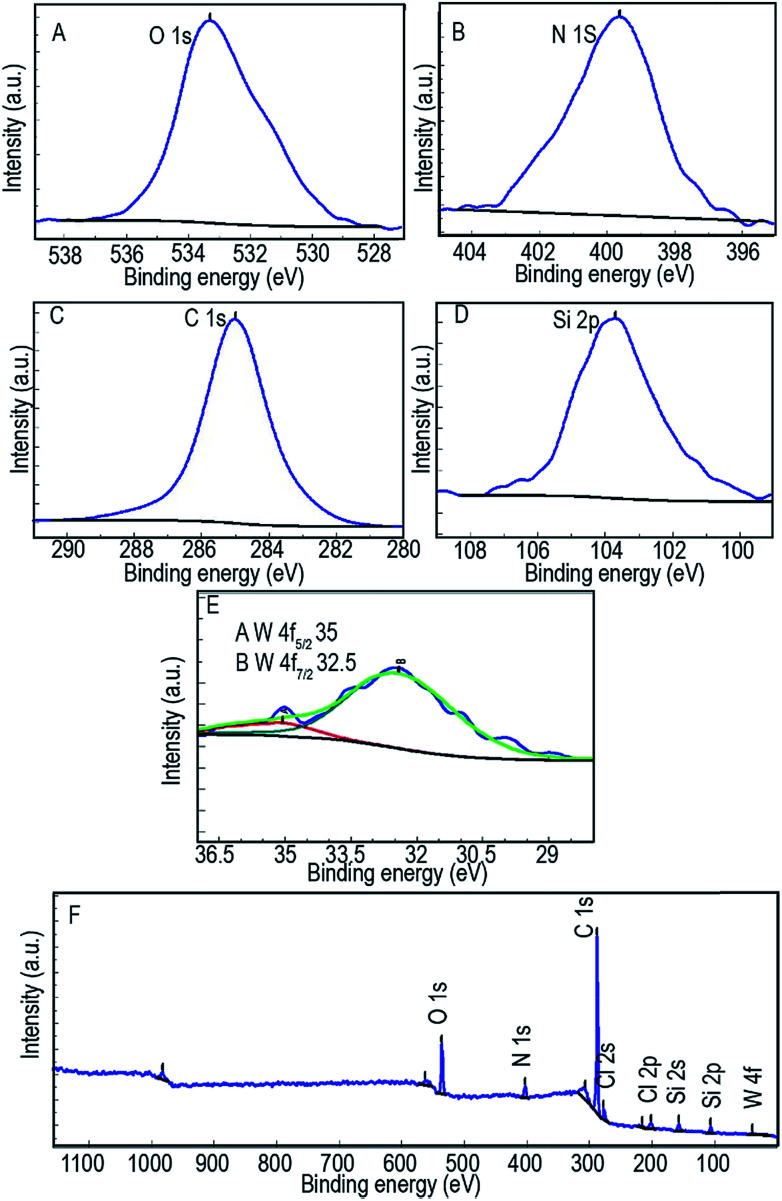
XPS spectra of (A) O 1s, (B) N 1s, (C) C 1s, (D) Si 2p, (E) W 4f and (F) W(iv)/NNBIA–SBA-15.

X-Ray photoelectron spectroscopy was applied to define the electronic state of the tungsten and the atomic concentration of the catalyst. The XPS spectrum of the catalyst shows peaks corresponding to Si, O, C 1s, N 1s, Cl 2s, Cl 2p, and W (Fig. 8F). The W 4f spectrum shows that the tungsten is present as W^4+^, with W 4f_5/2_ and W 4f_7/2_ binding energies of 35 and 33.5 eV, respectively, with an approximate intensity ratio of 3 : 4 (ref. 59) (Fig. 8E). The Si 2s and Si 2p peaks are centered at 103 and 150 eV respectively and clearly show the presence of the SiO_2_ structure^60^ (Fig. 8D). Fig. 8C shows a peak at 285.0 eV for C 1s to π*(C=C) and π*(C=N) orbitals.^61^ A broad peak extending from 396 to 403 eV is observed for all of the N contained within the catalyst (Fig. 8B). The O 1s XPS spectrum shown in Fig. 8A reveals a well-defined peak at 533.52 eV that could be attributed to SiO_2_ (533 eV), C–O (531–532 eV) and C=O (533 eV). Information on the atomic concentration of the W(iv)/NNBIA–SBA-15, as obtained from XPS, is summarized in Table 2.

**Table 2:** Results of XPS elemental analysis in % for W(iv)/NNBIA–SBA-15

Sample	O	N	C	Cl	Si	W
W(iv)/NNBIA–SBA-15 (%)	11.0	5.0	78.0	1.8	4.0	0.1

### 2.2. Catalytic tests

#### 2.2.1. Comparison of the efficiency of W(iv)/NNBIA–SBA-15 and other catalysts

The merit of the W(iv)/NNBIA–SBA-15 nanocatalyst was investigated in an MCR reaction. The reaction of isatin, malononitrile, and hydantoin was selected as a model reaction and the results were summarized in Table 3. Almost no corresponding product was obtained in the absence of catalyst after 48 h under reflux or at 80 °C in water. We compared the efficiency of homogeneous catalysts such as piperidine, triethylamine, l-proline, DABCO, and NH_2_–SBA-15 with W(iv)/NNBIA–SBA-15. The obtained results indicated that the presence of the nanocatalyst, W(iv)/NNBIA–SBA-15, was much more efficient than previously reported homogenous basic catalysts (Table 3, entries 3–7). The adsorption of reactants on the surface of the nanocatalyst support increased the local concentration of reactants around the active sites of W(iv)/NNBIA–SBA-15. The effect of functionalization was evaluated using SBA-15 alone. The results indicated that SBA-15 alone was almost inactive, which is probably due to the absence of active sites (Table 3, entries 7 and 8). The effect of the metal was investigated. Based on the results, the absence of metal in the catalyst decreased the yield and increased the time of the reaction (Table 3, entries 7 and 9). WCl_6_ alone provided the desired product in a 50% yield which points to the importance of the presence of the support and the ligand (Table 3, entries 7 and 10).

**Table 3:** The effect of the different catalysts on the synthesis of 2-azapyrrolizidine alkaloids

Entry	Catalyst^a^	Time (h)	Yield^b^ (%)
1	No catalyst (reflux)	48	Trace
2	No catalyst	48	Trace
3	Piperidine (20 mol%)	8	50
4	NEt_3_ (20 mol%)	7	75
5	l-Prolin (20 mol%)	15	32
6	DABCO (20 mol%)	8	17
7	W(iv)/NNBIA–SBA-15 (0.03 g)	3	95
8	SBA-15	18	≥20
9	NNBIA–SBA-15	18	40
10	WCl_6_	12	50

a Reactions conditions: isatin (1 mmol), malononitrile (1 mmol), and hydantoin (1 mmol) in water at 80 °C.

b Isolated yield.

#### 2.2.2. Effect of solvent, temperature and catalyst amount on the synthesis of 2-azapyrrolizidine alkaloids

The effect of water and organic solvents such as EtOH, PhCH_3_, CH_3_CN, and DMF were studied on the sequential one-pot reaction (Table 4, entries 1–6). In the presence of polar solvents such as H_2_O and EtOH, the product was synthesized in excellent yields, although a gummy solid was obtained with EtOH. The best results were obtained using water in terms of both yield and time. Thus we kept this as the optimal solvent for the subsequent reactions.

**Table 4:** Optimization of solvent, temperature and catalyst amount for the synthesis of 2-azapyrrolizidine alkaloids

Entry^a^	Solvent	Temp. (°C)	Catalyst amount (g)	Time (h)	Yield^b^ (%)
1	CH_3_CN	Reflux	0.03	12	72
2	CH_3_Ph	Reflux	0.03	48	Trace
3	DMF	Reflux	0.03	12	24
4	EtOH	Reflux	0.03	4	83
5	EtOH–water	Reflux	0.03	4	85
6	Water	Reflux	0.03	8	50
7	Water	r.t.	0.03	48	5
8	Water	80	0.03	3	95
9	Water	80	0.02	4	80
10	Water	80	0.04	4	95

a Reactions conditions: isatin (1 mmol), malononitrile (1 mmol), and hydantoin (1 mmol).

b Isolated yield.

The temperature had the greatest effect on the reaction progress. A trace amount of product was yielded at room temperature after 48 h, and the results of the reflux conditions were not appropriate (Table 4, entries 6–8). The best results were obtained at 80 °C.

The influence of the catalyst amount was studied under the optimum conditions (Table 4, entries 8–10). An acceptable reaction time and yield were obtained by loading 0.03 g of catalyst, and no benefit was observed with a greater catalyst amount.

#### 2.2.3. Synthesis of 2-azapyrrolizidine alkaloids derivatives in the presence of W(iv)/NNBIA–SBA-15 under optimized conditions

The crucial characteristics of the new organic–inorganic catalyst, W(iv)/NNBIA–SBA-15, are chemical stability, reusability, and non-toxicity. The efficiency of the catalyst was tested for the synthesis of a broad range of 2-azapyrrolizidine alkaloids. The methodology was explored by using various carbonyl derivatives (1 mmol), malononitrile (1 mmol) and hydantoin (1 mmol), under optimized conditions. In the presence of various isatin compounds, the desired products were obtained in good yield and diastereoselectivity (Table 5). Furthermore, the results of the scope of the reaction showed that the reaction progressed well with different aldehydes with electron donating and electron withdrawing groups substituted (Table 6).

**Table 5:** Synthesis of spirooxindole-2-azapyrrolizidine derivatives

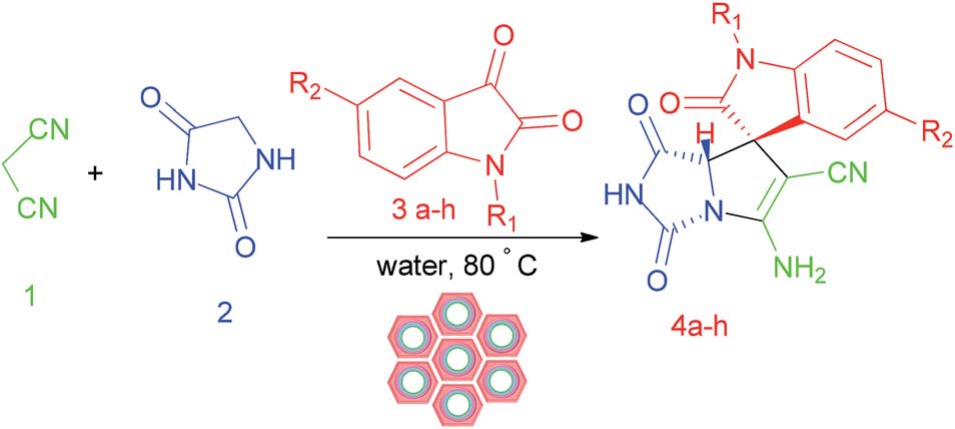						
Entry^a^	Isatin 1a–g		Product 4a–h	Time (h)	Yield^b^ (%)	mp (°C)
	*R*_1_	*R*_2_				
1	H	H	4a	3	95	260
2	Me	H	4b	4	85	280
3	H	Cl	4c	4	80	300
4	H	F	4d	5	79	304
5	H	Br	4e	5	90	318
6	H	I	4f	5	85	291
7	H	NO_2_	4g	6	78	340
8	H	OMe	4h	5	90	271

a Reactions conditions: isatin derivatives (1 mmol), malononitrile (1 mmol), and hydantoin (1 mmol).

b Isolated yield.

**Table 6:** Synthesis of 2-azapyrrolizidine derivatives

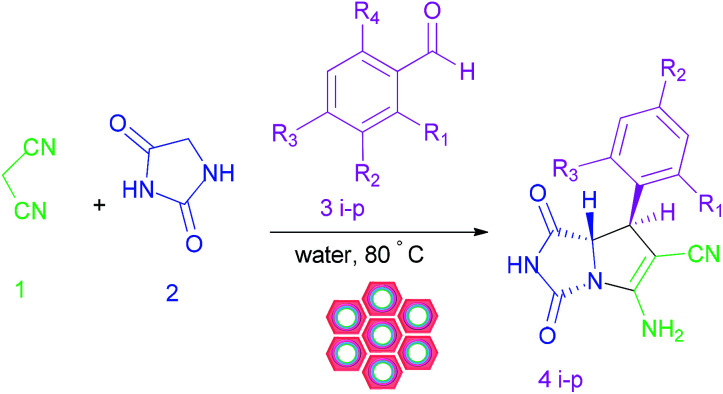								
Entry^a^	Aldehyde				Product	Time (h)	Yield^b^ (%)	mp (°C)
	*R*_1_	*R*_2_	*R*_3_	*R*_4_				
1	H	H	OMe	H	4i	3	95	260
2	H	H	Me	H	4j	3	95	260
3	H	H	H	H	4k	1	92	275
4	OMe	H	OMe	H	4l	6	50	245
5	H	H	Br	H	4m		74	271
6	H	NO_2_	H	H	4n	6	85	265
7	H		Cl	H	4o	2	96	281
8	Cl		H	Cl	4p	5	54	265

a Reactions conditions: aldehyde derivatives (1 mmol), malononitrile (1 mmol), and hydantoin (1 mmol).

b Isolated yield.

### 2.3. Characterization of the products of the three component reactions

**Scheme 2:**
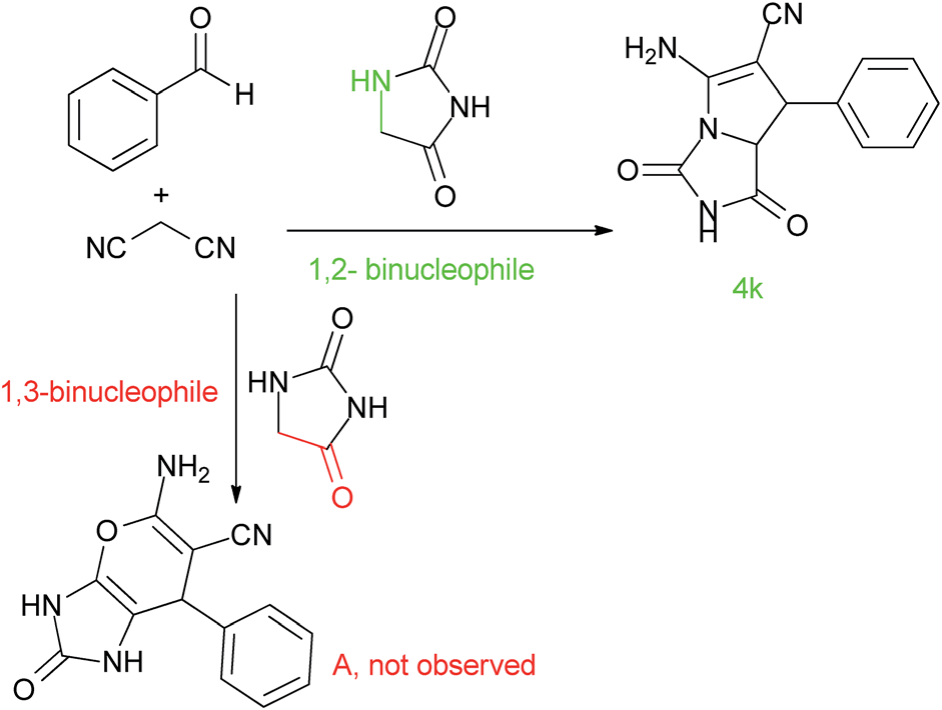
Investigation on the structure of hydantoin.

High diastereoselectivity can be observed by using aldehydes to produce only the *trans* diastereomer. The MCR reaction causes the generation of a *cis*-fused 2-azapyrrolizidine. Actually, the two hydrogens attached to the adjacent chiral carbon are *trans*. In the presence of isatin, a highly strained spirooxindole with a quaternary stereocenter was generated in only one diastereomer of a racemic mixture. The regioselectivity of the reaction can be rationalized by analysing the structure of hydantoin. Scheme 2 shows that hydantoin can play either the role of a 1,3-binucleophile when using the (CH_2_) and the (O) of the amide moiety or the role of a 1,2-binucleophile when using the (CH_2_) and the adjacent (NH). Pyrano-spirooxindole (A) is the corresponding product if hydantoin reacts as a 1,3-binucleophile and product 4k is expected if hydantoin reacts as a 1,2-binucleophile. Structure 4k was confirmed by ^1^H NMR spectroscopy. It indicates exchangeable protons at 11.67, 10.60 and 7.76 ppm with a 1 : 1 : 2 ratio, that can be referenced to two types of (NH) and an (NH_2_) respectively. In addition, the ^1^H NMR shows 1 proton for the one singlet at 5.11 ppm which is matched by the structure of 4k.

### 2.4. A plausible mechanism for the preparation of 2-azapyrrolizidine alkaloids

**Scheme 3:**
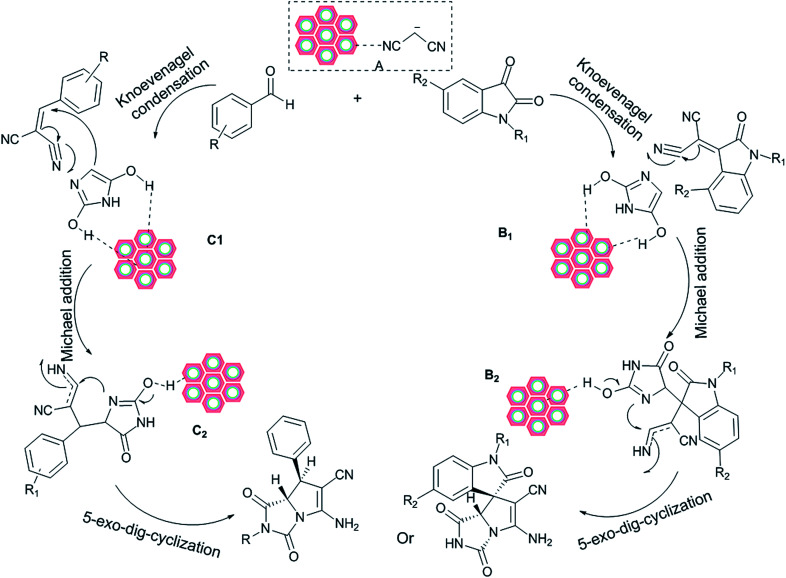
The proposed mechanism for the synthesis of azapyrrolizidine alkaloids.

A plausible catalytic cycle for the present reaction is illustrated in Scheme 3. The electrophilicity of malononitrile is increased by coordination with the W(iv)/NNBIA–SBA-15. The nitrile is coordinated to a tungsten site which is followed by a mechanism involving a proton abstraction, and a stable hydrido(enolato)-complex (A) is afforded.^62^ Then the Knoevenagel condensations of (A) with carbonyl group derivatives are carried out to produce the cyano olefins (B1) and (C1). Deprotonations of the cyano olefins (B1) and (C1) take place at the amino groups distributed on the surface of W(iv)/NNBIA–SBA-15 to yield the nucleophiles. On the other hand, coordination of the carbonyl groups of hydantoin to a tungsten site of the W(iv)/NNBIA–SBA-15 activates the electrophile in close proximity to the nucleophile. As the second step, a Michael addition occurs to generate intermediates (B2) and (C2).^63^ They are held in close proximity to the W(iv)/NNBIA–SBA-15 through coordination of the carbonyl groups of hydantoin to a tungsten site of the W(iv)/NNBIA–SBA-15. Then, the reaction might follow either a 5-*exo*-dig or 6-*exo*-dig pathway. However, the reactions are chemoselective and the corresponding products were achieved by 5-*exo*-dig. The amino groups distributed on the surface of W(iv)/NNBIA–SBA-15 provide a nucleophilic mechanism. Tungsten sites at the surface of the nanocatalyst increase the electrophilicity of the reactants. The high surface area of the SBA-15 is very important for the high turnover frequency of the reaction. The role of water as the solvent was evaluated. Water molecules form H-bonds with organic reactants that have hydrogen bond acceptor sites, both in their initial states and in their reaction transition states. H-bonding reduces the energy of the frontier orbitals by decreasing interorbital repulsion and electron density.^64^

### 2.5. Hot filtration test for the W(iv)/NNBIA–SBA-15 catalyst

We did a hot filtration test to prove that the organic moiety was attached to SBA-15 by covalent bonding that leads to stable attachment and less leaching. This test was carried out on the model reaction in the presence of 0.03 g of W(iv)/NNBIA–SBA-15 at 80 °C for 30 min. The reaction was continued for another 30 min in the absence of the catalyst and the yield of the product did not improve. Based on the results, the heterogeneity, the leaching-resistant properties and the stability of the W(iv)/NNBIA–SBA-15 catalyst have been confirmed.

### 2.6. Reusability of the W(iv)/NNBIA–SBA-15 catalyst

**Fig. 9:**
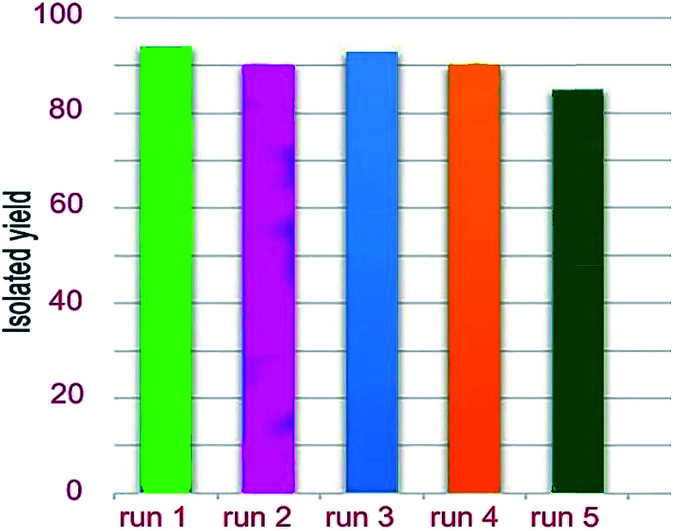
Recyclability study of the nanocatalyst, W(iv)/NNBIA–SBA-15, for the reaction of isatin (1 mmol), malononitrile (1 mmol) and hydantoin (1 mmol) under optimized conditions.

The model reaction with isatin (1 mmol), malononitrile (1 mmol), and hydantoin (1 mmol) was also employed to examine the reusability of the nanocatalyst, W(iv)/NNBIA–SBA-15. After the accomplishment of the reaction, the catalyst was filtered and washed with 5 mL hot ethanol (3 × 10 mL) and water (2 × 5 mL) and reused in a new reaction. However, in the second run, the conversion under similar conditions had a negligible reduction due to the blockage of catalytic sites with the reactants (Fig. 9). After the second run, the reused catalyst was washed three times with 5 mL hot ethanol (3 × 10 mL) and water (2 × 5 mL) to remove residual reactants and that resulted in the restoration of activity of the catalyst. In the third run, the reaction yield was improved by using the reused catalyst. Based upon these results, it was found that the W(iv)/NNBIA complex was strongly anchored on the mesoporous supports, and the catalytic activity did not decrease.

### 2.7. Study of the spectral data of some representative 2-azapyrrolizidine alkaloid scaffolds

#### 2.7.1 4a: (*trans*-3,7a′)-5′-amino-1′,2,3′-trioxo1′,2′,3′,7a′-tetrahydro spiro[indoline-3,7′-pyrrolo[1,2-*c*]imidazole]-6′-carbonitrile

mp = 260–262 °C; IR (KBr, cm^−1^) = 3372, 3341, 3252, 3200, 2182, 1791, 1738, 1702, 1656, 1620; ^1^H NMR (400 MHz, DMSO-d_6_): *δ* = 11.680 (s, 1H, NH), 10.609 (s, 1H, NH), 7.775 (br, s, 2H, NH_2_), 7.408 (d, *J* = 8 Hz, 1H), 7.253 (t, *J* = 8 Hz, 1H), 7.049 (t, *J* = 8 Hz, 1H), 6.853 (d, *J* = 8 Hz, 1H), 5.11 (s, 1H, CH); anal. calcd for C_14_H_9_N_5_O_3_: C, 56.45; H, 3.07; N, 23.72; found: C, 56.99; H, 3.12; N, 23.78; HR-MS *m*/*z*: calcd for C_14_H_9_N_5_O_3_ [M + Na]^+^: 318.0603; found: 318.0609.

#### 2.7.2 4b: (*trans*-3,7a′)-5′-amino-1-methyl-1′,2,3′-trioxo-1′,2′,3′,7a′-tetrahydrospiro[indoline-3,7′-pyrrolo[1,2-*c*]imidazole]-6′-carbonitrile

mp = 280 °C; IR (KBr, cm^−1^): 3552, 3501, 3210, 2928, 2187, 1790, 1742, 1688, 1648; ^1^H NMR (400 MHz, DMSO-d_6_): *δ* = 11.04 (br, 1H, NH), 7.741 (br, 2H, NH_2_), 7.526 (d, *J* = 7.5 Hz, 1H), 7.339 (t, *J* = 7.5 Hz, 1H), 7.108 (t, *J* = 7.5 Hz, 1H), 7.072 (d, *J* = 7.5 Hz, 1H), 5.094 (s, 1H, CH), 3.019 (s, 3H, CH_3_); anal. calcd for C_15_H_11_N_5_O_3_: C, 58.14; H, 3.72; N, 22.59; found: C, 58.02; H, 3.52; N, 22.21; HR-MS *m*/*z*: calcd for C_15_H_11_N_5_O_3_ [M + Na]^+^: 332.0759; found: 332.0762.

#### 2.7.3 4c: (*trans*-3,7a′)-5′-amino-5-chloro-1′,2,3′-trioxo-1′,2′,3′,7a′-tetrahydrospiro[indoline-3,7′-pyrrolo[1,2-*c*]imidazole]-6′-carbonitrile

mp = 300 °C; IR (KBr, cm^−1^): 3493, 3392, 3237, 2750, 2197, 1790, 1742, 1700, 1636, 1572; ^1^H NMR (400 MHz, DMSO-d_6_): *δ* = 11.057 (br, 1H, NH), 10.749 (br, 1H, NH), 7.831 (br, 2H, NH_2_), 7.568 (s, 1H), 7.333 (d, *J* = 7.2 Hz, 1H), 6.882 (d, *J* = 7.2 Hz, 1H), 5.168 (s, 1H, CH); C_14_H_8_ClN_5_O_3_: C, 51.12; H, 2.64; N, 21.32; found: C, 51.23; H, 2.34; N, 21.17; HR-MS *m*/*z*: calcd for C_14_H_8_ClN_5_O_3_ [M + Na]^+^: 352.0213; found: 352.0216.

#### 2.7.4 4d: (*trans*-3,7a′)-5′-amino-5-bromo-1′,2,3′-trioxo-1′,2′,3′,7a′-tetrahydrospiro[indoline-3,7′-pyrrolo[1,2-*c*]imidazole]-6′-carbonitrile

mp = 304 °C; IR (KBr, cm^−1^): 3485, 3385, 3243, 3150, 2736, 2193, 1790, 1744, 1707, 1634; ^1^H NMR (400 MHz, DMSO-d_6_): *δ* = 12.45 (br, 1H, NH), 11.127 (br, 1H, NH), 7.728 (br, 2H, NH_2_), 7.708 (d, *J* = 7.5 Hz, 1H), 7.288 (s, 1H), 6.865 (d, *J* = 7.5 Hz, 1H), 5.150 (s, 1H, CH); anal. calcd for C_14_H_8_BrN_5_O_3_: C, 44.82; H, 2.13; N, 18.61; found: C, 44.70; H, 2.53; N, 18.83. HR-MS *m*/*z*: calcd for C_14_H_8_BrN_5_O_3_ [M + Na]^+^: 395.9708; found: 395.9709.

#### 2.7.5 4e: (*trans*-3,7a′)-5′-amino-5-fluoro-1′,2,3′-trioxo-1′,2′,3′,7a′-tetrahydrospiro[indoline-3,7′-pyrrolo[1,2-*c*]imidazole]-6′-carbonitrile

mp = 318 °C; IR (KBr, cm^−1^) = 3378, 3200, 2186, 1794, 1712, 1662, 1601; ^1^H NMR (400 MHz, DMSO-d_6_): *δ* = 11.697 (s, 1H, NH), 10.643 (s, 1H, NH), 7.83 (br, 2H, NH_2_), 7.44 (d, *J* = 8.4, 1H), 7.16–7.11 (m, 1H), 6.88 (d, *J* = 8.4, 1H), 5.16 (s, 1H, CH); anal. calcd for C_14_H_8_FN_5_O_3_: C, 53.68; H, 2.57; N, 22.36; found: C, 53.52; H, 2.59; N, 22.42; HR-MS *m*/*z*: calcd for C_14_H_8_FN_5_O_3_ [M + Na]^+^: 336.0508; found: 336.0509.

#### 2.7.6 4f: (*trans*-3,7a′)-5′-amino-5-iodo-1′,2,3′-trioxo-1′,2′,3′,7a′-tetrahydrospiro[indoline-3,7′-pyrrolo[1,2-*c*]imidazole]-6′-carbonitrile

mp = 291 °C; IR (KBr, cm^−1^): 3375, 3346, 3258, 3204, 2192, 1798, 1743, 1712, 1660, 1623; ^1^H NMR (400 MHz, DMSO-d_6_): *δ* = 11.71 (s, 1H), 10.73 (s, 1H), 7.83 (br, s, 2H), 7.80 (d, *J* = 1.6 Hz, 1H), 7.63 (q, *J* = 8.4, 8.0 Hz, 1H), 5.18 (s, 1H); anal. calcd for C_14_H_8_IN_5_O_3_: C, 39.91; H, 3.37; N, 21.42; found: C, 40.01; H, 3.38; N, 21.39. HR-MS *m*/*z*: calcd for C_14_H_8_IN_5_O_3_ [M + Na]^+^: 443.9568; found: 443.9570.

#### 2.7.7 4g: (*trans*-3,7a′)-5′-amino-5-nitro-1′,2,3′-trioxo-1′,2′,3′,7a′-tetrahydrospiro[indoline-3,7′-pyrrolo[1,2-*c*]imidazole]-6′-carbonitrile

mp = 340 °C; IR (KBr, cm^−1^) = 3499, 3424, 3302, 3195, 2200, 1807, 1785, 1751, 1735, 1722, 1650, 1628; ^1^H NMR (400 MHz, DMSO-d_6_): *δ* = 11.648 (br, 1H, NH), 11.283 (br, 1H, NH), 8.440 (d, *J* = 8 Hz, 1H), 8.20 (s, 1H), 7.640 (br s, NH_2_), 7.066 (d, *J* = 8.Hz, 1H), 5.437 (s, 1H, CH); anal. calcd for C_14_H_8_N_6_O_5_: C, 49.41; H, 2.36; N, 24.69; found: C, 50.01; H, 2.48; N, 24.52. HR-MS *m*/*z*: calcd for C_14_H_8_N_6_O_5_ [M + Na]^+^: 363.0453; found: 363.0455.

#### 2.7.8 4h: (*trans*-3,7a′)-5′-amino-5-methoxy-1′,2,3′-trioxo-1′,2′,3′,7a′-tetrahydrospiro[indoline-3,7′-pyrrolo[1,2-*c*]imidazole]-6′-carbonitrile

mp = 265 °C; IR (KBr, cm^−1^): 3377, 3325, 3267, 2895, 2187, 1801, 1748, 1720, 1659, 1593; ^1^H NMR (400 MHz, DMSO-d_6_): *δ* = 11.67 (br, 1H, NH), 10.42 (br, 1H, NH), 7.76 (br, 2H, NH_2_), 7.10 (d, *J* = 8.4 Hz, 1H), 6.84 (d, *J* = 8.4, 1H), 6.77 (d, *J* = 8.4 Hz, 1H), 5.12 (s, 1H, CH), 3.73 (s, 3H, OCH_3_); anal. calcd for C_15_H_11_N_5_O_4_: C, 55.28; H, 3.37; N, 21.42; found: C, 55.39; H, 3.38; N, 21.52. HR-MS *m*/*z*: calcd for C_15_H_11_N_5_O_3_ [M + Na]^+^: 348.0639; found: 348.0642.

#### 2.7.9 4i: (*trans*-7,7a)-5-amino-7-(4-methoxyphenyl)-1,3-dioxo-2,3,7,7a-tetrahydro-1*H*-pyrrolo[1,2-*c*]imidazole-6-carbonitrile

mp = 260 °C, IR (KBr, cm^−1^) = 3390, 3340, 3258, 3210, 2183, 1775, 1713, 1662, 1596, ^1^H NMR (400 MHz, DMSO-d_6_): *δ* = 9.248 (s, NH), 7.308 (br s, NH_2_), 7.221 (d, *J* = 8 Hz, 2H), 6.894 (d, *J* = 8 Hz, 2H), 4.473 (s, 2H), 3.739 (s, 3H) ppm, ^1^H-NMR (300 MHz, DMSO-d_6_ with a drop of D_2_O): *δ* = 7.192 (d, *J* = 8 Hz, 2H), 6.926 (d, *J* = 8 Hz, 2H), 4.454 (d, *J* = 7.5 Hz, 1H), 4.379 (d, *J* = 7.5 Hz, 1H) 3.690 (s, 3H). If DMSO was used as a solvent for ^1^H NMR spectroscopy, a singlet peak was revealed for two adjacent protons, which implied a low amount of Δ*ν* in the DMSO solvent. But in the presence of D_2_O, two doublet peaks appeared that had *J* = 7.5 Hz. It can be inferred from these results that just the *trans* diastereomer, confirmed by X-Ray crystallography, was the pure product; anal. calcd for C_14_H_12_N_4_O_3_: C, 59.15, H, 4.25, N, 19.70; found: C, 59.23, H, 4.38, N, 19.41. HR-MS *m*/*z*: calcd for C_14_H_12_N_4_O_3_ [M + Na]^+^: 307.0807; found: 307.0808.

#### 2.7.10 4j: (*trans*-7,7a)-5-amino-1,3-dioxo-7-(*p*-tolyl)-2,3,7,7a-tetrahydro-1*H*-pyrrolo[1,2-*c*]-imidazole-6-carbonitrile

mp = 275 °C; IR (KBr, cm^−1^) = 3400, 3318, 3260, 2169, 1778, 1708, 1653, 1598; ^1^H NMR (400 MHz, DMSO-d_6_): *δ* = 8.46 (s, 1H, NH), 7.862 (d, *J* = 8 Hz, 2H), 7.438 (d, *J* = 8 Hz, 2H), 7.313 (br s, NH_2_), 4.448 (s, 2H, CH_2_); 2.389 (s, 3H); anal. calcd for C_14_H_12_N_5_O_4_: C, 62.69; H, 4.50; N, 20.88; found: C, 62.73; H, 4.22; N, 20.52; HR-MS *m*/*z*: calcd for C_14_H_12_N_5_O_4_ [M + Na]^+^: 291.0858; found: 291.0859.

#### 2.7.11 4k: (*trans*-7,7a)-5-amino-1,3-dioxo-7-phenyl-2,3,7,7a-tetrahydro-1*H*-pyrrolo[1,2-*c*]-imidazole-6-carbonitrile

mp = 275 °C; IR (KBr, cm^−1^): 3400, 3318, 3260, 2169, 1778, 1708, 1653, 1598; ^1^H NMR (400 MHz, DMSO-d_6_): *δ* = 8.504 (br s, NH), 7.313 (br s, NH_2_), 7.110–7.115 (m, 5H, Ar–H), 4.428 (s, 2H, CH_2_); anal. calcd for C_13_H_10_N_4_O_2_: C, 61.41, H, 3.96; N, 22.03; found: C, 61.29; H, 3.88; N, 22.22. HR-MS *m*/*z*: calcd for C_13_H_10_N_4_O_2_ [M + Na]^+^: 277.0701; found: 277.0705.

#### 2.7.12 4l: (*trans*-7,7a)-5-amino-7-(3,5-dimethoxyphenyl)-1,3-dioxo-2,3,7,7a-tetrahydro-1*H*-pyrrolo[1,2-*c*]imidazole-6-carbonitrile

mp = 245 °C; IR (KBr, cm^−1^): 3402, 3339, 3241, 3010, 2967, 2838, 2771, 2195, 1744, 1661; ^1^H NMR (400 MHz, DMSO-d_6_): *δ* = 9.815 (br s, NH), 8.323 (br s, NH_2_), 7.608 (s, 1H), 7.591 (d, *J* = 8.0 Hz, 1H), 7.201 (d, *J* = 8.0 Hz, 1H), 4.636 (d, *J* = 8 Hz, 2H), 3.889 (d, *J* = 8 Hz, 6H); anal. calcd for C_15_H_14_N_4_O_4_: C, 57.32; H, 4.48; N, 17.82; found: C, 57.29; H, 4.58; N, 17.79. HR-MS *m*/*z*: calcd for C_15_H_14_N_4_O_4_ [M + Na]^+^: 337.0912; found: 337.0915.

#### 2.7.13 4m: (*trans*-7,7a)-5-amino-7-(4-bromophenyl)-1,3-dioxo-2,3,7,7a-tetrahydro-1*H*-pyrrolo[1,2-*c*]imidazole-6-carbonitrile

mp = 271 °C; IR (KBr, cm^−1^): 3391, 3313, 3259, 3209, 2182, 1782, 1718, 1645, 1623; ^1^H NMR (400 MHz, DMSO-d_6_): *δ* = 11.49 (s, 1H), 7.59 (d, *J* = 8.4 Hz, 2H), 7.39 (br s, 2H), 7.30 (d, *J* = 8.4 Hz, 2H) 4.57 (d, *J* = 9.2 Hz, 1H) 4.54 (d, *J* = 9.2 Hz, 1H) ppm; anal. calcd for C_13_H_9_BrN_4_O_2_: C, 46.85, H, 2.72; N, 16.82; found: C, 46.92; H, 2.69; N, 16.72; HR-MS *m*/*z*: calcd for C_13_H_9_BrN_4_O_2_ [M + Na]^+^: 354.9806; found: 354.9809.

#### 2.7.14 4n: (*trans*-7,7a)-5-amino-7-(3-nitrophenyl)-1,3-dioxo-2,3,7,7a-tetrahydro-1*H*-pyrrolo[1,2-*c*]-imidazole-6-carbonitrile

mp = 265 °C; IR (KBr, cm^−1^): 3444, 3347, 3218, 2180, 1789, 1751, 1655; ^1^H NMR (400 MHz, DMSO-d_6_): *δ* = 11.55 (s, 1H), 8.21–7.70 (m, 4H), 7.51 (br s, 2H), 4.79 (d, *J* = 8.8 Hz, 1H), 4.66 (d, *J* = 8.8 Hz, 1H); anal. calcd for C_13_H_9_N_5_O_4_: C, 52.16, H, 3.03; N, 23.41; found: C, 51.92; H, 2.96; N, 23.72; HR-MS *m*/*z*: calcd for C_13_H_9_N_5_O_4_ [M + Na]^+^: 322.0552; found: 322.0554.

#### 2.7.15 4o: (*trans*-7,7a)-5-amino-7-(4-chlorophenyl)-1,3-dioxo-2,3,7,7a-tetrahydro-1*H*-pyrrolo[1,2-*c*]imidazole-6-carbonitrile

mp = 281 °C; IR (KBr, cm^−1^): 3394, 3318, 3268, 3217, 3181, 2193, 1783, 1723, 1660, 1603; ^1^H NMR (400 MHz, DMSO-d_6_): *δ* = 7.748 (br s, NH), 7.464 (d, *J* = 8.4 Hz, 2H), 7.255 (br s, NH_2_), 6.957 (d, *J* = 8.4 Hz, 2H), 4.58 (s, 2H); anal. calcd for C_13_H_9_ClN_4_O_2_: C, 54.08; H, 3.14; N, 12.28; found: C, 54.29; H, 3.21; N, 12.22; HR-MS *m*/*z*: calcd for C_13_H_9_ClN_4_O_2_ [M + Na]^+^: 311.0311; found: 311.0313.

#### 2.7.16 4p: (*trans*-7,7a)-5-amino-7-(2,6-dichlorophenyl)-1,3-dioxo-2,3,7,7a-tetrahydro-1*H*-pyrrolo-[1,2-*c*]imidazole-6-carbonitrile

mp = 265 °C; IR (KBr, cm^−1^): = 3432, 3340, 3227, 2182, 1791, 1712, 1659, 1602 cm^−1^; ^1^H NMR (400 MHz, DMSO-d_6_): *δ* = 11.46 (br s, NH), 7.45–7.27 (m, 3H), 7.31 (br s, NH_2_), 5.13 (d, *J* = 8.3 Hz, 1H), 4.85 (s, 1H); anal. calcd for C_13_H_8_Cl_2_N_4_O_2_: C, 48.32; H, 2.49, N, 21.94; found: C, 48.29, H, 2.21; N, 21.81; HR-MS *m*/*z*: calcd for C_13_H_8_Cl_2_N_4_O_2_ [M + Na]^+^: 344.9922; found: 344.9925.

## 3. Experimental section

All starting materials and solvents were purchased commercially from Merck and Sigma-Aldrich and were used as received. The ^1^H NMR spectra were determined using Bruker Avance-400 MHz spectrometers with DMSO-d_6_ as a solvent and tetramethylsilane (TMS) as an internal standard. FT-IR measurements were carried out using a Magna 550 apparatus by using KBr plates. The elemental analyses (C. H. N) of the samples were recorded using a LECO CHNS 923 analyzer. The XRD patterns were evaluated using an X-ray diffractometer (PHILIPS, PW 1510, Netherlands) with Cu-Kα radiation (*λ* = 0.154056 nm) in the range 2*θ* = 0–10°. The TGA-DTA analyses were performed using a Bahr STA-503 instrument in air at a heating rate of 10 °C min^−1^. The N_2_ adsorption analysis was recorded at −196 °C using an automated gas adsorption analyzer (BEL SORP mini II) and the pore diameter was calculated from the adsorption branch of the isotherm by using the BJH model. The FESEM analysis of the nanoparticles was carried out using a Model FE-SEM. The TEM imaging analysis was performed using a Philips EM208 transmission electron microscope with an accelerating voltage of 200 kV. The EDX analysis of the nanoparticles was carried out using a Sigma ZEISS, Oxford Instruments Field Emission. In the NH_3_-TPD experiments, samples were pre-treated at 573 K for 1 h. After that, NH_3_ was adsorbed at 373 K for 0.5 h. Then, physically adsorbed ammonia was removed with a pure nitrogen flow at 373 K. XPS spectra were recorded using a ESCA System 100 spectrometer (VSW Scientific Instruments, Manchester).

### 3.1. Preparation and characterization of the catalyst

**Scheme 4:**
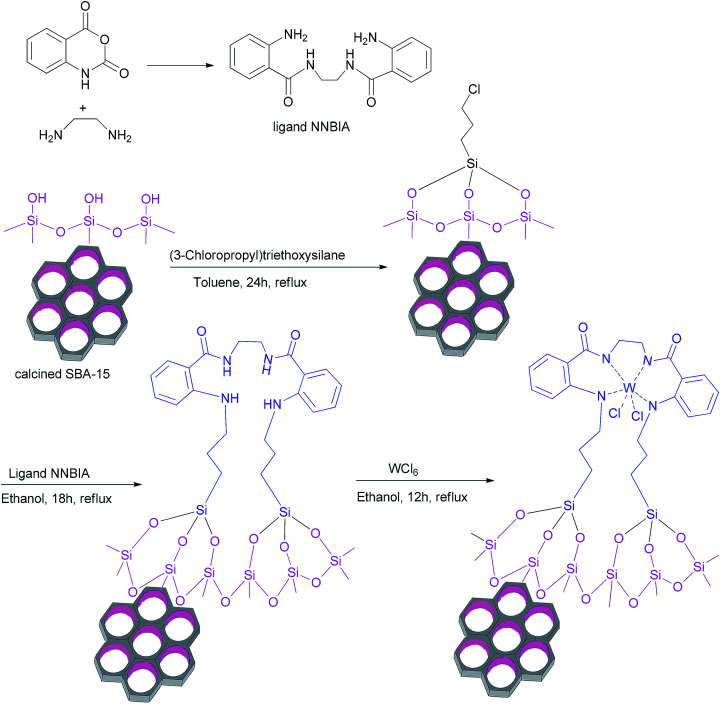
The different steps for synthesis of W(iv)/NNBIA–SBA-15.

All of the steps for the functionalization of the modified surface of the mesoporous SBA-15 have been shown in Scheme 4.

### 3.2. Preparation of the NNBIA ligand

**Fig. 10:**
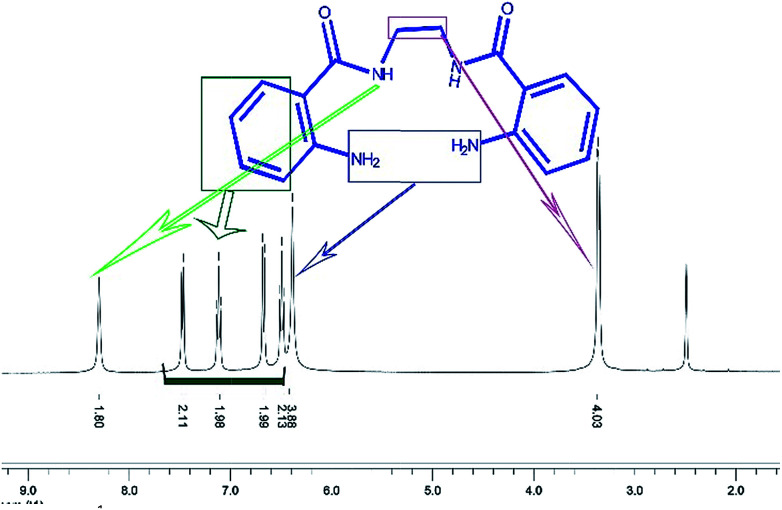
^1^H NMR spectrum of the ligand, NNBIA, in DMSO-d_6_.

1 mmol of 1,2-diaminoethane was added to a mixture of 2 mmol powdered isatoic anhydride in 5 mL of hot water. The reaction was stirred and refluxed for 3 h. Then, the reaction mixture was left to stand overnight to generate crystals of the product. The crystals were filtered and washed with EtOH and then dried to obtain the pure product. The structure of the ligand, NNBIA, was confirmed by the ^1^H NMR that is presented in Fig. 10.

### 3.3. Preparation of SBA-15

4.0 g of pluronic 123 triblock copolymers (EO_20_–PO_70_–EO_20_), as templates, were dissolved in 2.0 (M) aq. HCl (120 mL) and distilled water (15 mL) in a 250 mL round-bottom flask. The mixture was stirred at room temperature for 4 h. Then 8.5 g of tetraethylorthosilicate was added dropwise to the solution. The temperature of the reaction mixture was kept at 35 °C and it was stirred for 8 h. The synthesized gel was heated under static conditions in a Teflon-lined autoclave for 24 h at 100 °C. A white solid precipitated and was filtered and washed with deionized water. The residual organic materials were removed by calcination at 550 °C for 8 h.^42,48^

### 3.4. Organofunctionalization of SBA-15

In a dry Schlenk flask, 1 g of SBA-15, that was activated under vacuum at 150 °C for 3 h, was suspended in dry toluene. Then, 3 mmol of (3-chloropropyl)triethoxysilane was added to the above suspension solution. The reaction mixture was refluxed under nitrogen for 6 h. Finally, purification of the Cl-functionalized SBA-15 was achieved by Soxhlet extraction with dichloromethane for 8 h.

### 3.5. Immobilization of the NNBIA ligand over SBA-15–Cl

The SBA-15 with chloropropyl linkers (1 g) was suspended in absolute ethanol (60 mL) by sonication in a 250 mL round-bottom flask to form a uniform dispersion. Then, 0.298 g (1 mmol) of the NNBIA ligand was added to the solution and the reaction mixture was refluxed for 18 h. The precipitates were washed repeatedly with toluene to remove the unreacted substrate. Then, they were put in a hot-air oven at 90 °C and were left to stand overnight to dry and furnish the functionalized SBA-15.

### 3.6. Synthesis of W(iv)/NNBIA–SBA-15

1 g of functionalized SBA-15 were suspended in 50 mL of absolute ethanol. Then, 0.2 g of WCl_6_ was added and the mixture was refluxed for 12 h to anchor the W(iv). The green light solid that was W(iv)/NNBIA–SBA-15, resulted. It was washed repeatedly with ethanol and dried under vacuum.

### 3.7. General procedure for the preparation of the 2-azapyrrolizidine scaffold

In a 50 mL round-bottom flask, 1 mmol of isatin or aldehyde, 1 mmol of malononitrile, and 0.03 g of W(iv)/NNBIA–SBA-15 were mixed in 5 mL water. The mixture was stirred for 20 min at room temperature. Then hydantoin, as the third component, was added to the above mixture. The reaction temperature was increased to 80 °C. The mixture was stirred for the appropriate time that was founded by TLC. When the reaction was over, the precipitates were collected. They were dissolved in acetone and filtered to separate the catalyst. Acetone was evaporated and the product was purified by washing (2 × 10 mL) with a mixture of (3 : 1) ethyl acetate and hexane. The desired pure products were characterized by IR, ^1^H NMR and C. H. N. The W(iv)/NNBIA–SBA-15 could catalyze all of the reactions regardless of the substrates. The catalyst was washed three times with hot ethanol (3 × 10 mL) and water (2 × 5 mL), respectively.

## 4. Conclusions

A new functionalized mesoporous SBA-15 material has been introduced using a newly synthesized tetradentate ligand. The NNBIA ligand has many functional groups to increase the active site of the catalyst. The efficiency of the catalyst was evaluated by synthesizing 2-azapyrrolizidine alkaloids that have many biological properties. The one-pot reactions of isatin or aldehyde, malononitrile and hydantoin were accomplished in water as the solvent at 80 °C for the appropriate time. Some of the principles of green chemistry are followed in this synthetic method. We synthesized a wide range of 2-azapyrrolizidine alkaloids in very high yields by loading a very low amount of catalyst in the absence of any hazardous organic solvents. The benefits of this catalyst are easy reusability and high recyclability. Based on the results, the suggested method has an important role in developing the synthesis of 2-azapyrrolizidine alkaloids.

## Conflicts of interest

There are no conflicts to declare.

## Supplementary Material

RA-009-C9RA02825K-s001
