# Supplementary material for: Tungsten anchored onto functionalized SBA-15: an efficient catalyst for diastereoselective synthesis of 2-azapyrrolizidine alkaloid scaffolds
Source: RSC Adv. 2019 Jun 24;9(34):19662–74. doi: 10.1039/c9ra02825k (PMC9065583; doi:10.1039/c9ra02825k)
Supplement: RA-009-C9RA02825K-s001 [file RA-009-C9RA02825K-s001.pdf]

Electronic Supplementary Material (ESI) for RSC Advances.
This journal is © The Royal Society of Chemistry 2019

Tungsten anchored onto functionalized SBA-15: An efficient catalyst for diastereoselective synthesis of 2-azapyrrolizidine alkaloid scaffolds

Javad Safaei-Ghomi* and Atefeh Bakhtiari

Department of Organic Chemistry, Faculty of Chemistry, University of Kashan, Kashan, Iran. Fax: 98 591 2397; Tel: 98 591 2385;

E-mail: Safaei@kashanu.ac.ir

^{1}H NMR Spectra of compounds

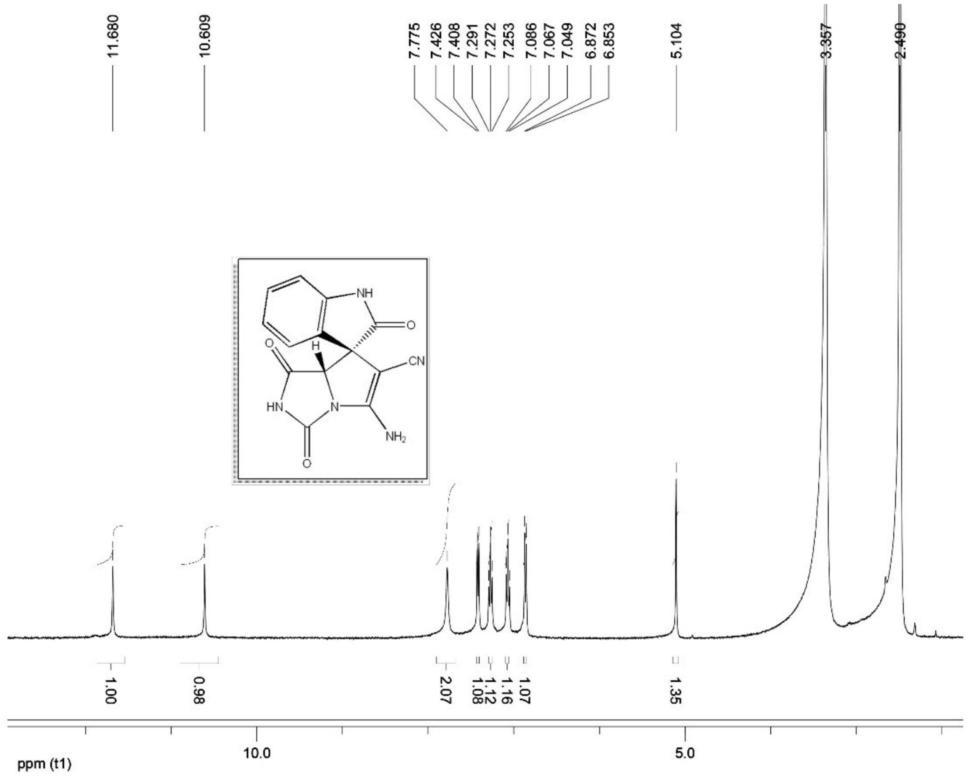

Figure S1. ^{1}H-NMR (400 MHz, DMSO-d$_6$) spectrum of compound 4a.

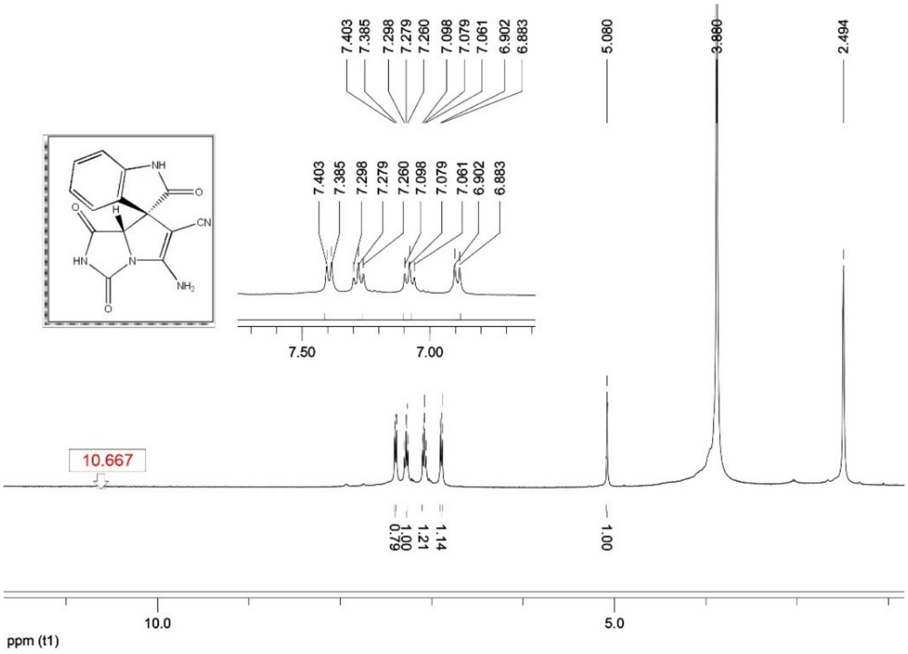

Figure S2. ^{1}H-NMR (400 MHz, DMSO-d_6 with a drop of D_2O) spectrum of compound 4a.

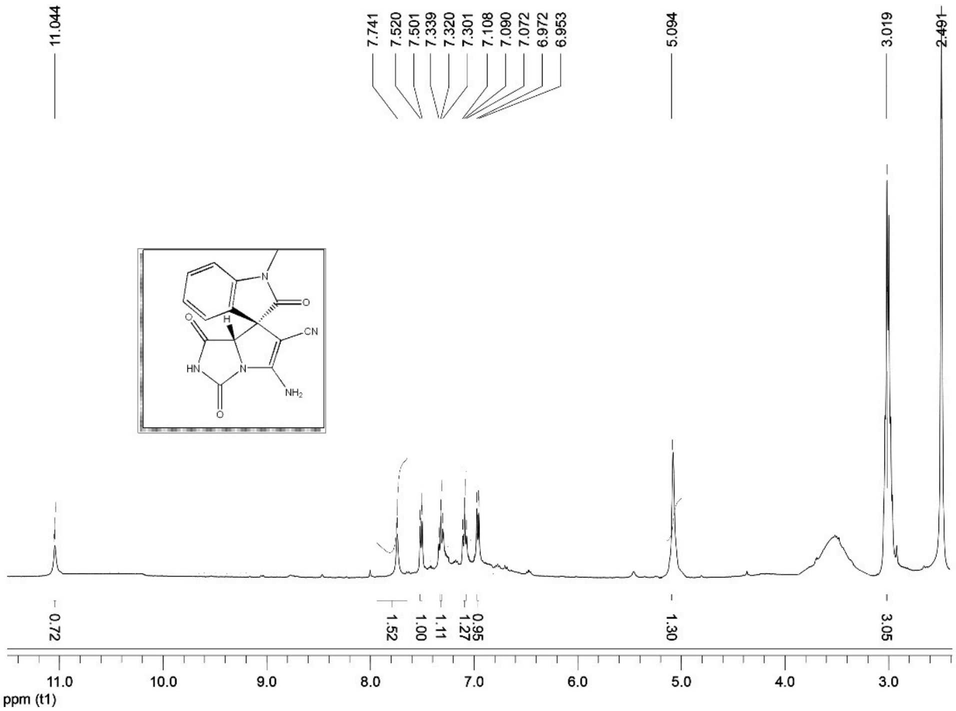

Figure S3. ^{1}H-NMR (400 MHz, DMSO-d_6) spectrum of compound 4b.

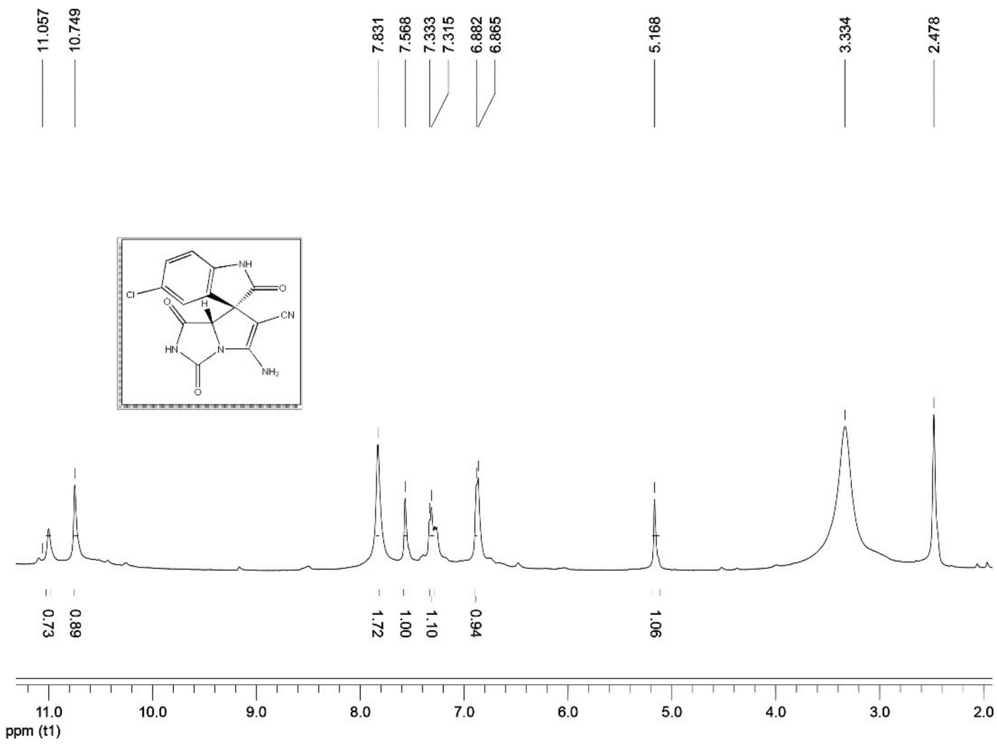

Figure S4. ^{1}H-NMR (400 MHz, DMSO-d_6) spectrum of compound 4c.

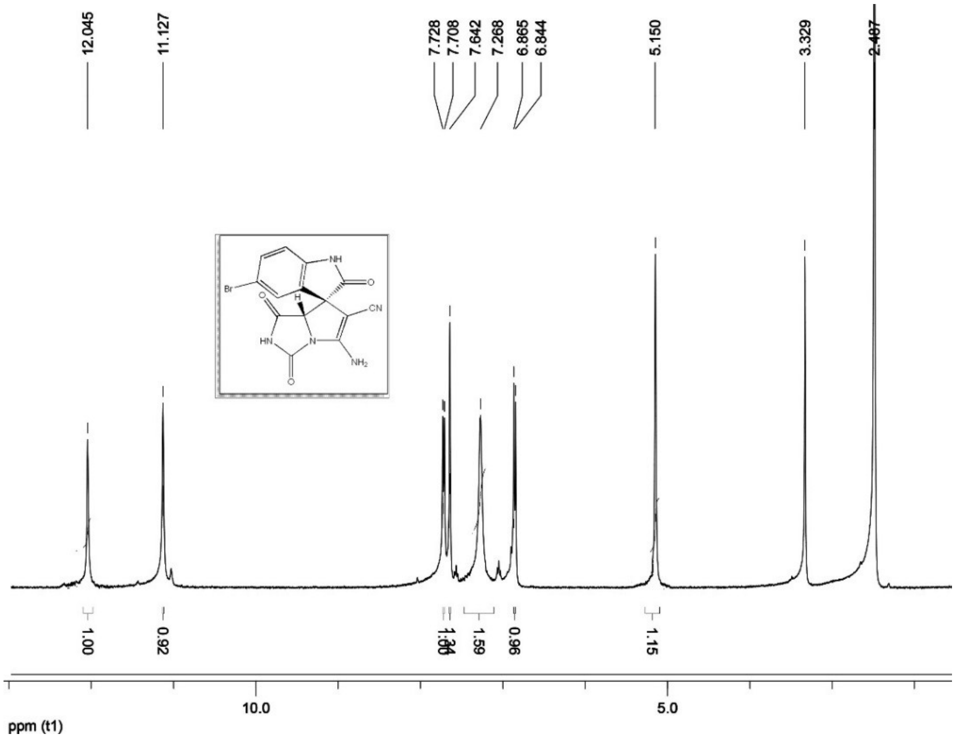

Figure S5. ^{1}H-NMR (400 MHz, DMSO-d_6) spectrum of compound 4d.

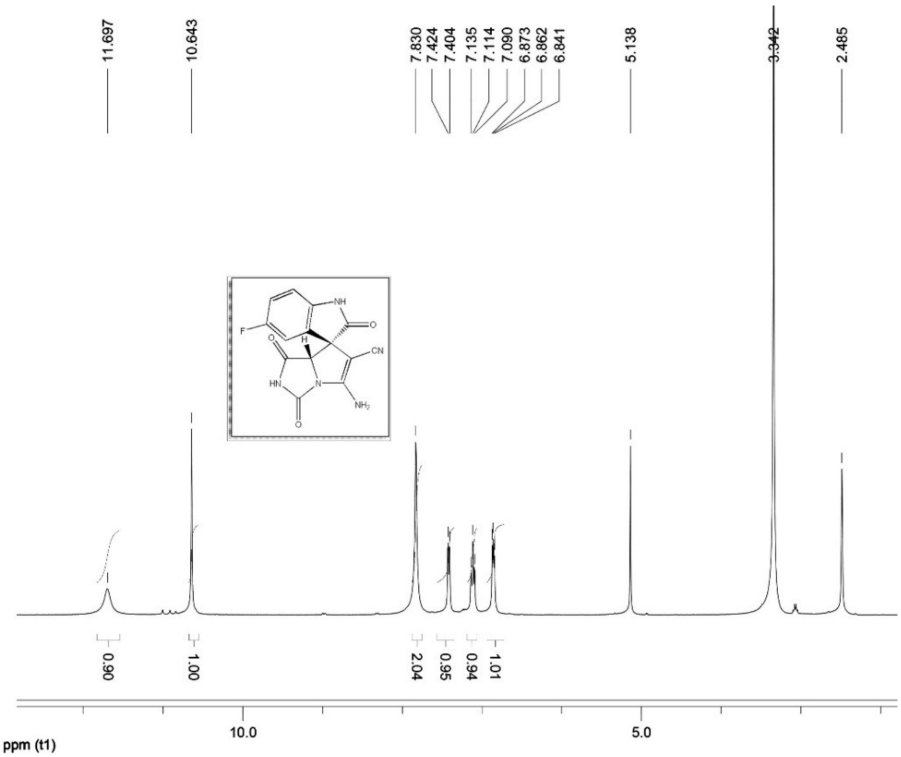

Figure S6. ^{1}H-NMR (400 MHz, DMSO-d_6) spectrum of compound 4e.

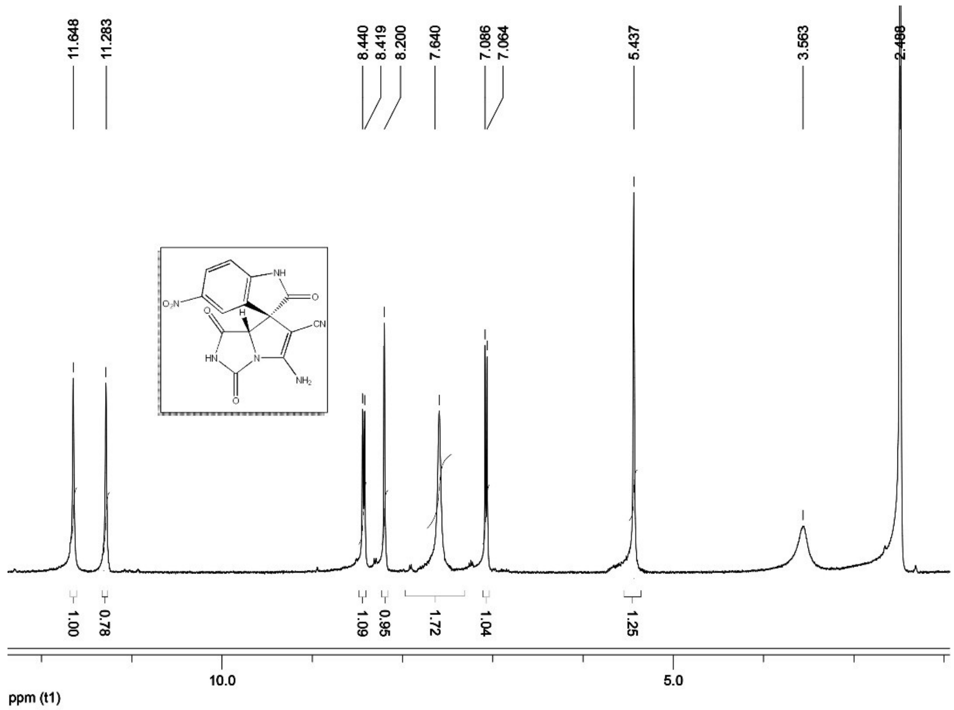

Figure S7. ^{1}H-NMR (400 MHz, DMSO-d_6) spectrum of compound 4g.

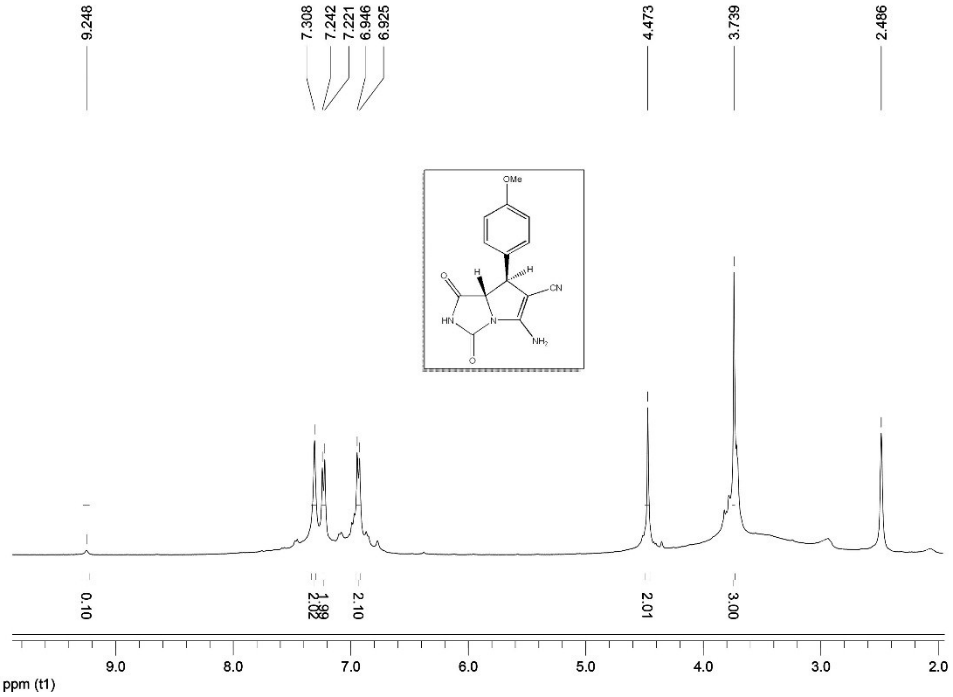

Figure S8. ^{1}H-NMR (400 MHz, DMSO-d_6) spectrum of compound 4i.

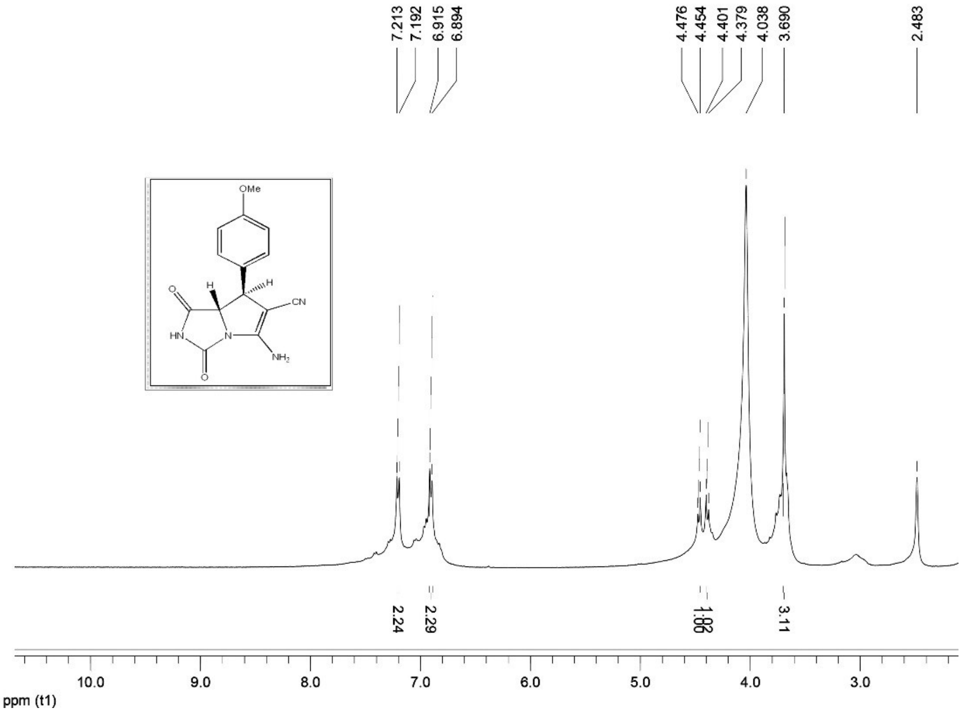

Figure S9. ^{1}H-NMR (400 MHz, DMSO-d_6 with a drop of D_2O) spectrum of compound 4i.

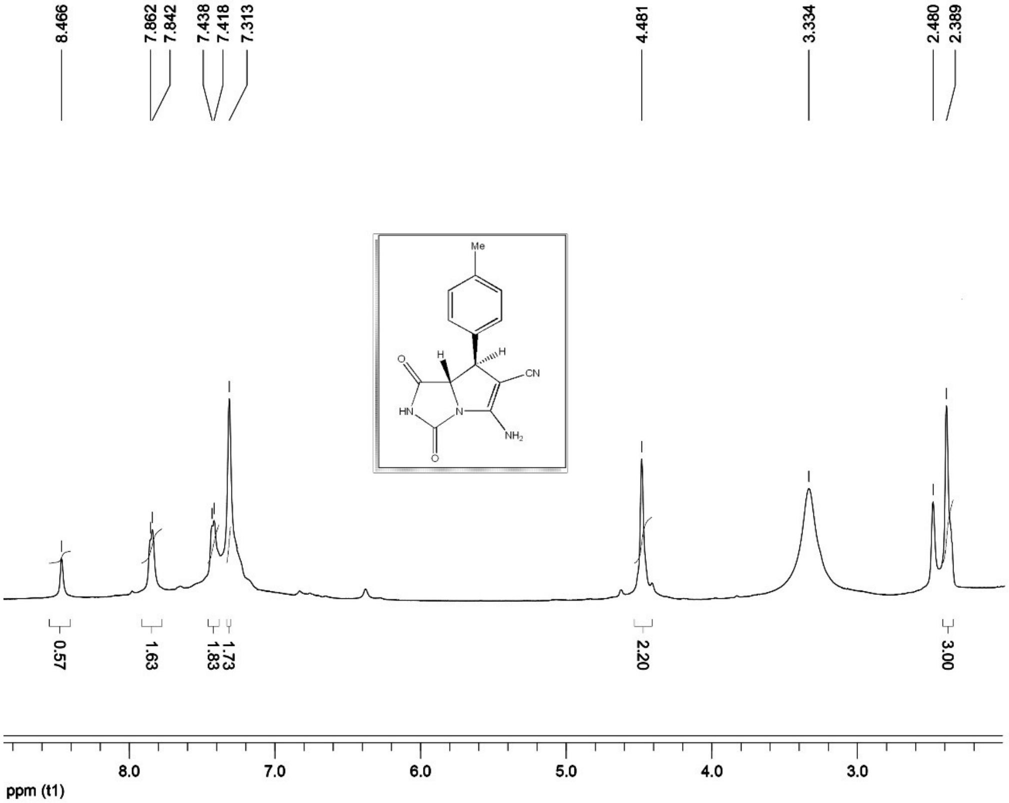

Figure S10. ^{1}H-NMR (400 MHz, DMSO-d$_6$) spectrum of compound 4j.

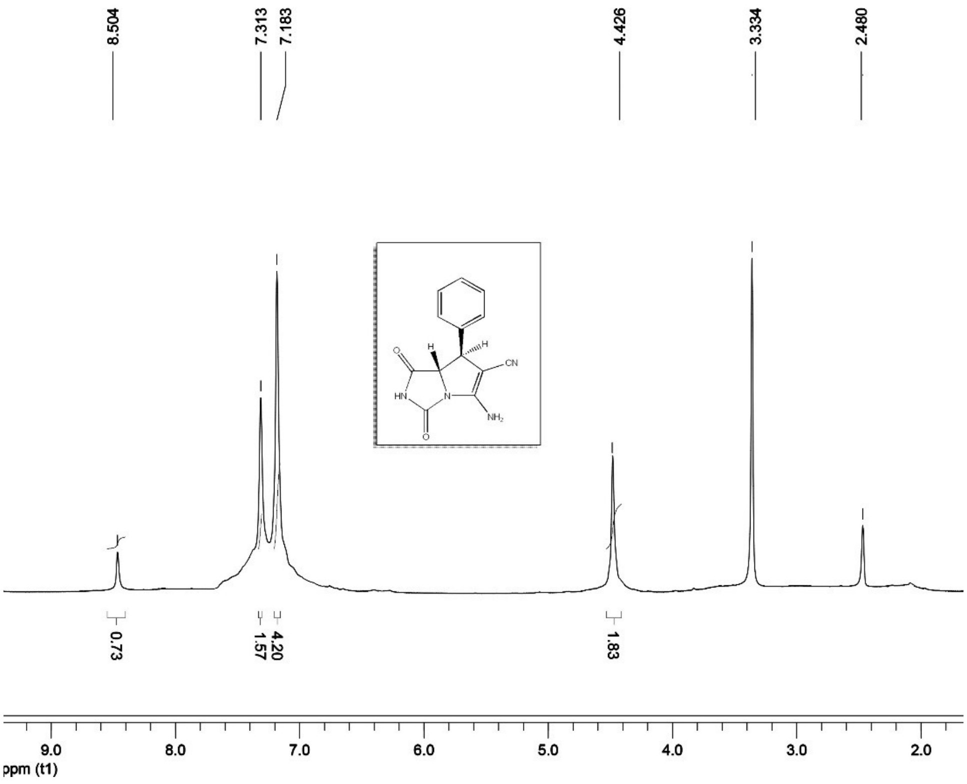

Figure S11. ^{1}H-NMR (400 MHz, DMSO-d_6) spectrum of compound 4k.

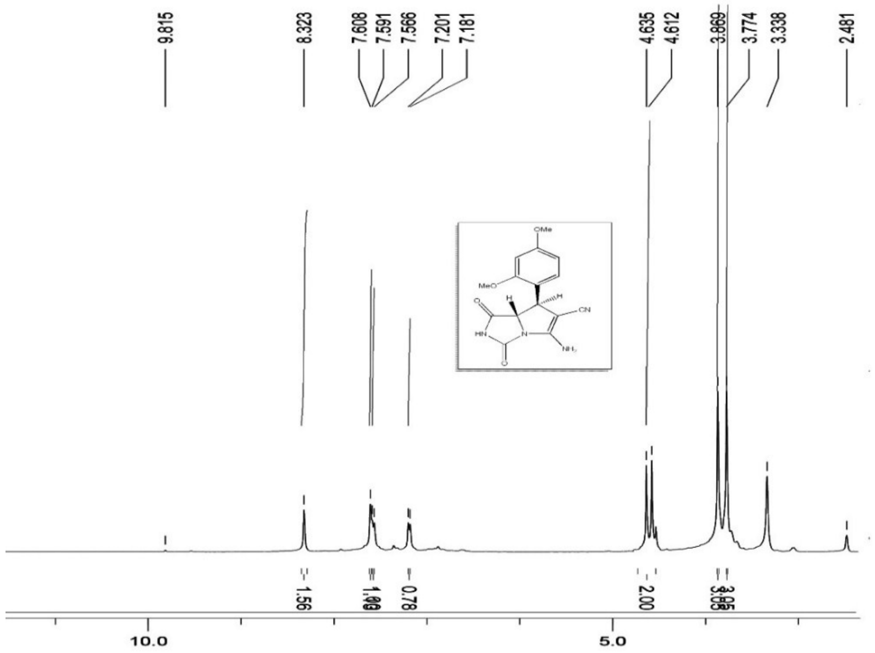

Figure S12. ^{1}H-NMR (400 MHz, DMSO-d_6) spectrum of compound 4l.

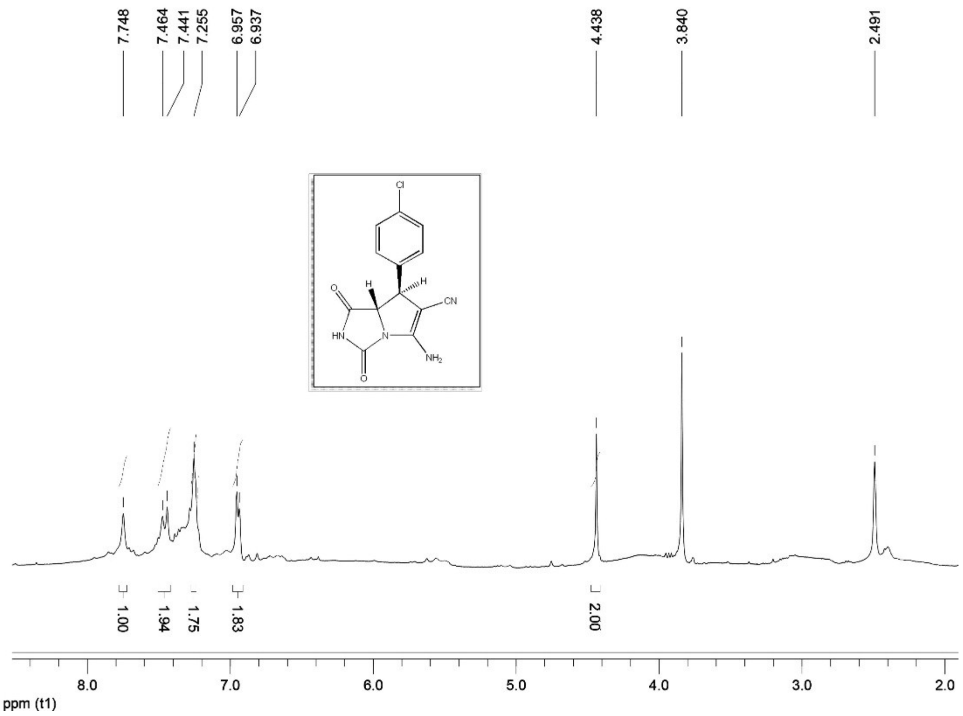

Figure S13. ^{1}H-NMR (400 MHz, DMSO-d_6) spectrum of compound 4o.
